# Hetero-Diels–Alder reactions of hetaryl and aryl thioketones with acetylenic dienophiles

**DOI:** 10.3762/bjoc.11.63

**Published:** 2015-04-28

**Authors:** Grzegorz Mlostoń, Paulina Grzelak, Maciej Mikina, Anthony Linden, Heinz Heimgartner

**Affiliations:** 1 Department of Organic and Applied Chemistry, University of Łódź, Tamka 12, PL 91-403 Łódź, Poland; 2 Center of Molecular and Macromolecular Studies PAS, Sienkiewicza 112, PL 90-363 Łódź, Poland; 3 Department of Chemistry, University of Zürich, Winterthurerstrasse 190, CH-8057 Zürich, Switzerland

**Keywords:** dimethyl acetylenedicarboxylate (DMAD), hetero-Diels–Alder reactions, high pressure reactions, methyl propiolate, thioketones, thiopyrans

## Abstract

Selected hetaryl and aryl thioketones react with acetylenecarboxylates under thermal conditions in the presence of LiClO_4_ or, alternatively, under high-pressure conditions (5 kbar) at room temperature yielding thiopyran derivatives. The hetero-Diels–Alder reaction occurs in a chemo- and regioselective manner. The initially formed [4 + 2] cycloadducts rearrange via a 1,3-hydrogen shift sequence to give the final products. The latter were smoothly oxidized by treatment with *m*CPBA to the corresponding sulfones.

## Introduction

**Scheme 1:**
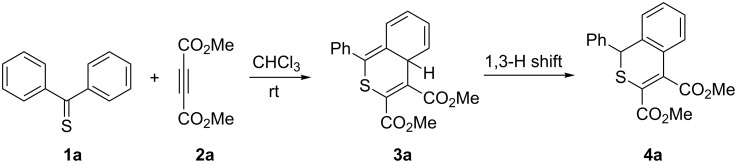
Hetero-Diels–Alder reaction of thiobenzophenone (**1a**) with dimethyl acetylenedicarboxylate (**2a**) [10].

A series of recent publications evidence that, in contrast to earlier opinions, thioketones are useful building blocks for the preparation of diverse sulfur heterocycles [1–3]. Studies performed by Huisgen and coworkers are of special importance and they resulted in the formulation of the name ‘superdipolarophiles’ for aromatic thioketones [4–6]. In addition, Sauer and coworkers called them ‘superdienophiles’ based on kinetic studies [7–8]. Moreover, thiobenzophenone (**1a**) was reported to react as a heterodiene smoothly with cyclooctyne, dicyanoacetylene, and dimethyl acetylenedicarboxylate (**2a**) to give [4 + 2] cycloadducts of type **3a**, which spontaneously rearrange via a 1,3-hydrogen shift yielding rearomatized products of type **4a** (Scheme 1) [9–11].

The same transformation occurred faster under photolytic conditions [9,12]. The hetero-Diels–Alder reaction of 4-substituted analogues of **1a** with in situ generated benzyne is also known [13]. Heteroaromatic thioketones are reported to undergo a hetero-Diels–Alder reaction with dienophiles such as maleic anhydride, acrylonitrile, styrene, and α-chloroacrylonitrile [14–15]. In the latter case, the stabilization of the initially formed cycloadduct occurred via HCl elimination, whereas, in the other cases, the 1,3-hydrogen shift led to rearomatized products.

In our ongoing studies on thioketones, we reported recently on selected reactions of new symmetrical and non-symmetrical hetaryl thioketones [16]. Among others, the reactions of **2a** with phenyl thien-2-yl thioketone as well as with bis(thien-2-yl) thioketone were described. The goal of the present study is to examine the reactions of diverse hetaryl thioketones with both **2a** and methyl propiolate (**2b**). Moreover, along with standard procedures, the high-pressure technique was applied. Finally, selected examples of the obtained polycyclic 2*H*-thiopyrans were oxidized to give the corresponding sulfones.

## Results and Discussion

Under standard conditions (benzene, rt), the reaction of **1a** with **2a** is slow, and it requires several days to be complete [9]. For that reason, we modified the procedure by using THF as a solvent and LiClO_4_ as a known, efficient catalyst applied frequently in diverse Diels–Alder reactions [17]. Heating the mixture in a closed tube to 50 °C resulted in completion of the reaction after only 24 h, and after chromatographic work-up, the known product **4a** was obtained in 84% yield. Another attempt to optimize the reaction conditions was based on the use of the high-pressure method. This approach offers some advantages, especially in the case of cycloaddition reactions [18]. To the best of our knowledge, reactions of thioketones under high-pressure conditions have never been studied. After a series of optimization experiments, a solution of **1a** and **2a** in a molar ratio of 1:2 in toluene was placed in a high-pressure vessel at 5 kbar, and after 24 h at room temperature, the product **4a** was isolated in 70% yield.

**Scheme 2:**
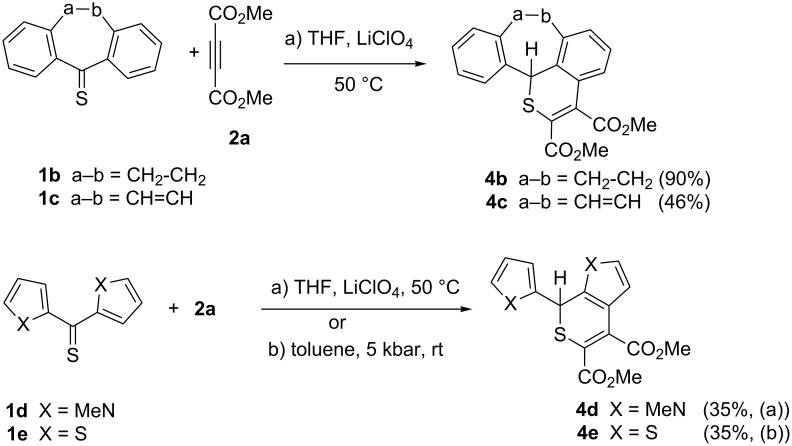
Synthesis of polycyclic thiopyrans via the hetero-Diels–Alder reaction/1,3-hydrogen shift sequence.

**Figure 1:**
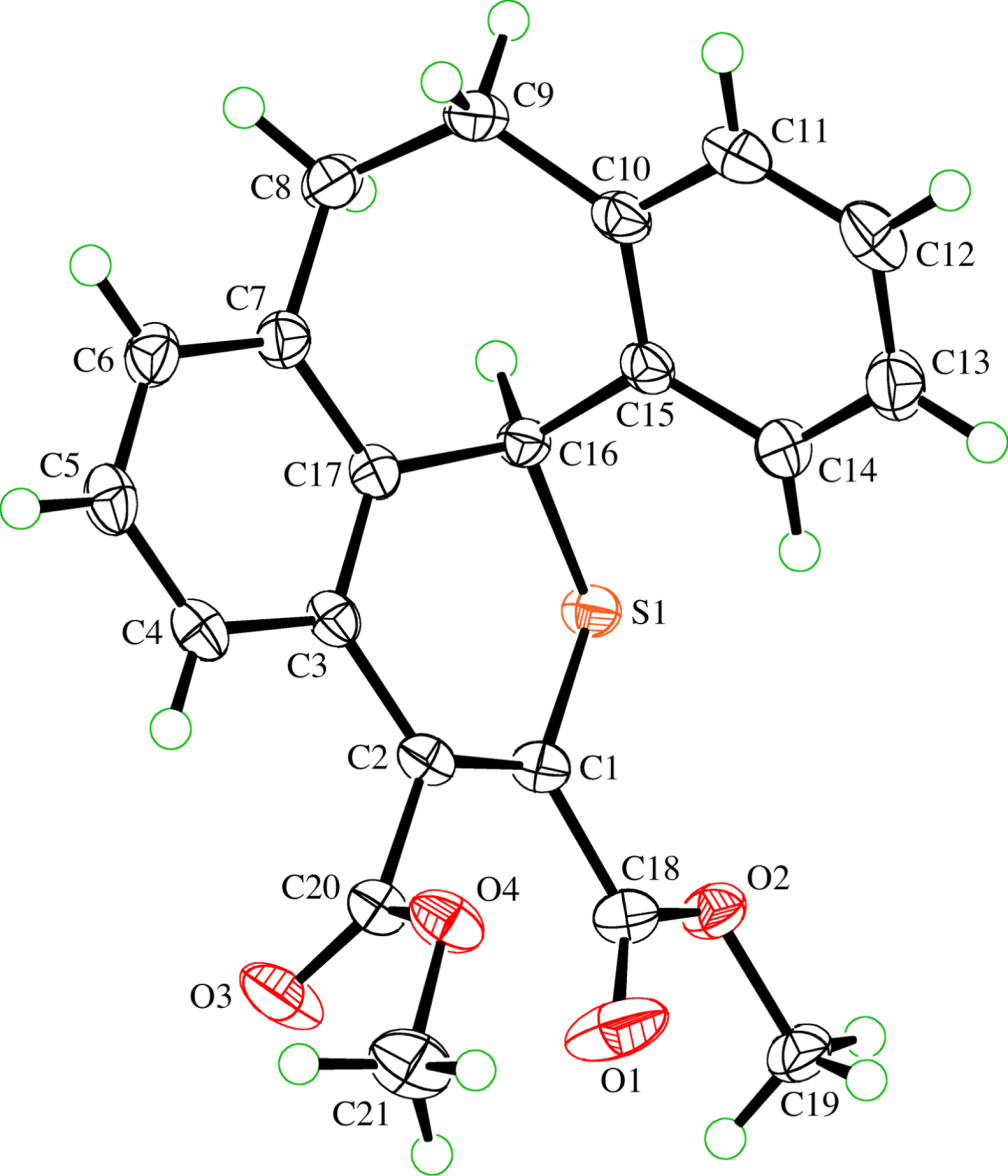
ORTEP Plot [19] of the molecular structure of **4b**, drawn using 50% probability displacement ellipsoids.

The reaction conditions with THF and LiClO_4_ were used for further reactions of aryl thioketones, i.e., thiodibenzosuberone **1b** and thiodibenzosuberenone **1c**, with **2a**. The expected polycyclic thiopyran derivatives **4b** and **4c**, respectively, were obtained in 90 and 46% yield (Scheme 2). In the ^1^H NMR spectra of both compounds the low-field shifted CHS signal appeared at 5.82 and 4.49 ppm. Finally, the 2*H*-thiopyran structure of **4b** was established by X-ray single crystal structure determination (Figure 1).

The analogous reactions of **2a** with bis(*N*-methylpyrrol-2-yl) thioketone (**1d**) afforded the pyrrolo[2,3-*c*]thiopyran derivative **4d** in modest yield (35%) (Scheme 2). In the case of bis(thien-2-yl) thioketone (**1e**), the reaction with **2a** was carried out in toluene at room temperature under a pressure of 5 kbar. After 72 h, the corresponding thieno[2,3-*c*]thiopyran **4e** was obtained in 35% yield (Scheme 2).

**Scheme 3:**
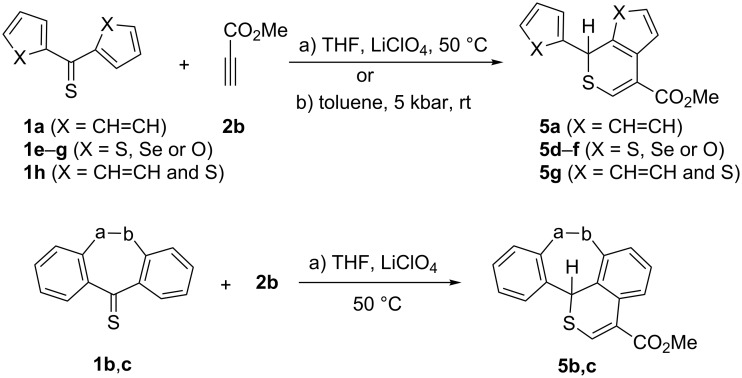
Reactions of aryl/hetaryl thioketones with methyl propiolate (Table 1).

In an extension of the study, reactions of methyl propiolate (**2b**) with selected aryl and hetaryl thioketones were performed under thermal and high-pressure conditions. The reaction of **1a** with **2b** in THF and a catalytic amount of LiClO_4_ led to the benzothiopyran **5a** in 61% yield (Scheme 3, Table 1). The ^1^H NMR analysis of the crude mixture indicated the presence of a single regioisomer for which the structure **5a** is proposed. In the alternative preparation of this product under high-pressure (toluene, 5 kbar), 66% of **5a** were isolated. Both methods were applied for analogous reactions with the symmetrical bis-hetaryl thioketones **1e–g**. Under thermal conditions, the products **5d**–**f** were also formed regioselectively in 83, 5, and 6% yield, respectively. In these cases, the high-pressure experiments were also performed with **1e** (16% yield of **5d**) and **1f** (33% yield of **5e**), respectively; thioketone **1g** was not tested under high pressure. However, attempted isolations of both **5e** and **5f** were unsuccessful as the products underwent decomposition upon chromatographic work-up. The experiment performed with the non-symmetrical phenyl thien-2-yl thioketone (**1h**) under thermal conditions resulted also in the formation of a single product **5g** in a chemo- and regioselective manner in 74% yield.

**Table 1:** Fused *2H*-thiopyrans **5**.

**1**	Ph/Hetar	Hetar/Ph	**5**	Method A^a^ yield [%]	Method B^a^ yield [%]

**a**	Ph	Ph	**a**	61	66
**b**	dibenzosuberone		**b**	94	–
**c**	dibenzosuberenone		**c**	61	–
**e**	thien-2-yl	thien-2-yl	**d**	83	16
**f**	selenophen-2-yl	selenophen-2-yl	**e**	5^b^	33^c^
**g**	furan-2-yl	furan-2-yl	**f**	6^b^	–
**h**	Ph	thien-2-yl	**g**	74	17

^a^Methods A and B see Experimental; ^b^not isolated; ^c^decomposes during attempted chromatographic separation.

The experiment under high-pressure conditions was much less successful and gave **5g** in only 17% yield. The structures of the products were determined based on the spectroscopic data. Thus, similar to hetero-Diels–Alder reactions with maleic anhydride [14], the C=C bond of the thiophene ring of **1h** is part of the reactive heterodiene system.

The reactions of **2b** with thiobenzosuberone **1b** and thiobenzosuberenone **1c** were performed under thermal conditions, and in both cases, new polycyclic thiopyran derivatives **5b** and **5c**, respectively, were formed regioselectively and obtained in good yields (94 and 61%, respectively).

In order to test the scope and limitations of the hetero-Diels–Alder reaction with thioketones and acetylenic dienophiles, other easily available acetylenes were used in the reaction with **1a**. In all reactions attempted with phenylacetylene, (pyridin-2-yl)acetylene, and (*tert*-butyl)acetylene, no conversion of **1a** was observed as evidenced by the blue color of the reaction mixture even after 24 h. Unfortunately, the experiments performed under the described conditions (THF, LiClO_4_, temp.) with (trifluoromethyl)acetylene and (diethoxyphosphoryl)acetylene, activated by the presence of strongly electron-withdrawing substituents, were also unsuccessful.

**Scheme 4:**
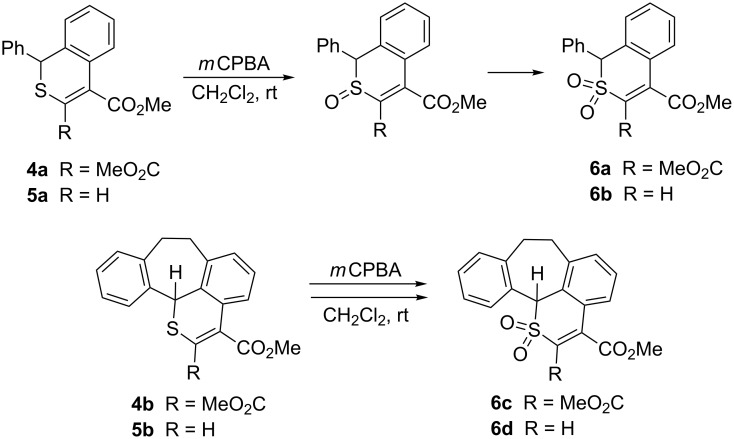
Oxidation of selected thiopyrans **4** and **5** to give the corresponding sulfones.

Some of the thiopyran derivatives **4** and **5** obtained in the present study were used for oxidation reactions aimed at the preparation of the corresponding sulfoxides and sulfones. As demonstrated in a recent publication [20], polycyclic sulfones are attractive substrates for the synthesis of polycyclic hydrocarbons via thermal SO_2_ extrusion. In our experiments, thiopyrans **4a**,**b** and **5a**,**b** were oxidized in CH_2_Cl_2_ solution at room temperature using 3.0 equivalents of *m*CPBA. In the case of **4a**, the progress of the reaction was monitored by TLC and ^1^H NMR spectroscopy. The spectrum recorded after 3 h showed that along with starting materials, two new products in a ratio of ca. 4:6 are present in the mixture. For that reason, the reaction time was extended to 3 days. Then, only one of these products was present, with characteristic signals of 2 MeO groups at 3.93 and 4.05 ppm. The signal of the CHS group was shifted downfield and appeared at 5.48 ppm. The ESI mass spectra as well as the elemental analysis confirmed the molecular formula C_19_H_16_SO_6_, which corresponds with the structure of the sulfone **6a** obtained in 90% yield (Scheme 4). Based on this result, the intermediate product observed in the mixture after 3 h can be proposed as the corresponding sulfoxide. No attempts were made to isolate this product.

**Figure 2:**
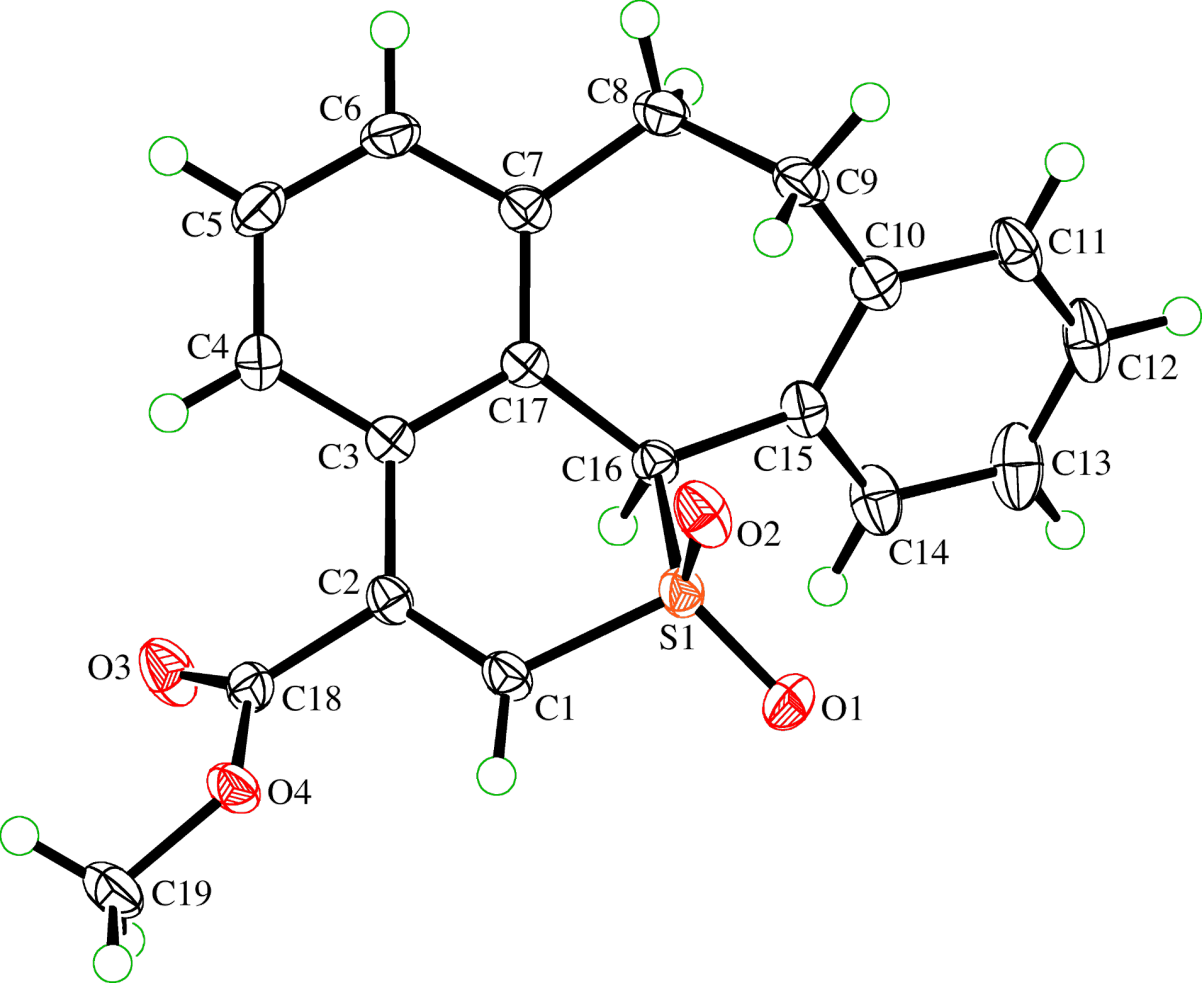
ORTEP Plot [19] of the molecular structure of one of the symmetry-independent molecules of **6d**, drawn using 50% probability displacement ellipsoids.

The same oxidation protocol was applied to convert thiopyranes **5a**, **4b**, and **5b** into the corresponding sulfones **6b**–**6d** in 94, 70, and 80% yield, respectively. The structure of **6d** was established by X-ray single crystal structure determination (Figure 2). The course of the oxidation reactions for these thiopyrans differs from a similar process reported for Se-containing systems. In these cases, ring contraction and elimination of an aryl group, but no formation of an oxidized product, was observed [21]. The same report describes the appearance of rearranged products (Pummerer-type rearrangement) upon treatment of 1*H*-2-benzothiopyran-3,4-dicarboxylates of type **4** with an equimolar amount of *m*CPBA.

## Conclusion

The present study shows that hetaryl thioketones react with activated acetylenecarboxylates in a hetero-Diels–Alder reaction followed by the 1,3-hydrogen shift to give fused thiopyran derivatives in a chemo- and regioselective manner. The thermal reaction can be catalyzed by LiClO_4_. Comparable reaction times were observed when the reaction was performed under high-pressure conditions at room temperature. The thiopyran derivatives can be oxidized by treatment with *m*CPBA at room temperature to give the corresponding sulfones. In this reaction, even when using equimolar amounts of *m*CPBA, selective formation of the sulfoxide could not be achieved. The obtained sulfones are promising substrates for thermal SO_2_ extrusion reactions aimed at the contraction of the ring. The importance of the presented study is amplified by the fact that benzothiopyran and related systems containing a thiopyran moiety, and especially their carboxylic derivatives, are known as important pharmacophores, with key importance for the biological activity of diverse sulfur polyheterocycles [22–24].

It is worth of mentioning, that the described hetero-Diels–Alder reactions with aromatic thioketones as heterodienes, display certain similarities to the reported organometallic pathways observed in their reactions with triiron dodecacarbonyl Fe_3_(CO)_12_ [25–26].

## Experimental

**General information:** Melting points were determined in capillaries using a MEL-TEMP II apparatus (Aldrich) and are uncorrected. IR spectra were recorded with a FTIR NEXUS spectrophotometer as KBr pellets; absorptions (ν) in cm^−1^. ^1^H and ^13^C NMR spectra were measured on a Bruker Avance III (^1^H at 600 and ^13^C at 150 MHz) instrument in CDCl_3_; chemical shifts (δ) are given in ppm, coupling constants (*J*) in Hz. The multiplicity of the ^13^C signals was deduced from DEPT, supported by ^1^H,^13^C HMQC spectra. ^1^H NMR data are presented as follows: chemical shift, multiplicity (br = broad, s = singlet, d = doublet, t = triplet, q = quartet, m = multiplet), coupling constant, integration. The mass spectra were recorded on a Finnigan MAT-95 (ESI), Bruker maxis (HRESI), or SYNAPT G2-S HMDS (HR MALDI–TOF) instrument. Elemental analyses were performed in the Microanalytical Laboraory of the Chemistry Faculty in Łódź. Reactions under high pressure were performed in a High Pressure Autoclave LC10T in the Laboratory of the Polish Academy of Sciences (CBMiM) in Łódź. Applied reagents such as DMAD, methyl propiolate, inorganic reagents, and solvents are commercially available (Aldrich) and were used as received.

**Reaction of thioketones 1a–h with acetylenic dienophiles 2a or 2b – General procedures: Method A:** A solution of 1 mmol of the corresponding thioketone, 2 mmol of the corresponding acetylenic dienophiles and 10 mol % of LiClO_4_ in 1 mL of dry THF was placed in a thick-wall glass tube, which then was closed with a screw cap. The mixture was heated at 50 °C for 24 h. The solvent was evaporated in vacuo. Subsequent separation on preparative plates coated with silica gel (eluent: CH_2_Cl_2_) gave pure products. **Method B:** The corresponding thioketone (1 mmol) and acetylenic dienophile (2 mmol) were placed in a 4 mL teflon vial that was then filled with dry toluene. The vial was closed and kept at 5 kbar at room temperature for 24 h. After depressurizing, the solvent was removed in vacuo. Subsequent separation on preparative plates coated with silica gel (eluent: CH_2_Cl_2_) gave pure products.

**Dimethyl 11,12-dihydro-4b*H*-benzo[4,5]cyclohepta[1,2,3-*ij*]isothiochromene-6,7-dicarboxylate (4b):** Yield: 328.5 mg (90%). Colorless crystals; mp 147.5–148.0 °C (MeOH); IR (KBr) ν: 3061 (w), 2948 (w), 1732 (s), 1725 (s), 1597 (w), 1562 (w), 1435 (m), 1270 (s), 1232 (s), 1030 (w), 751(m) cm^−1^; ^1^H NMR (600 MHz, CDCl_3_) δ 7.61 (d, *J* = 12 Hz, 1H_arom_), 7.33–7.30 (m, 1H_arom_), 7.24–7.15 (m, 4H_arom_), 7.03 (d, *J* = 6 Hz, 1H_arom_), 5.82 (br s, S-CH), 3.92, 3.94 (2s, 6H, 2 OCH_3_), 3.55 (br s, 1CH), 3.37 (br s, 1CH), 3.12 (br s, 1CH), 3.01–2.97 (m, 1CH) ppm; ^13^C NMR (150 MHz, CDCl_3_) δ 168.1, 164.3 (2 C=O), 138.1, 138.0, 131.8, 130.6 (7 C(sp^2^)), 136.3, 128.0, 127.5, 126.4, 125.3 (7 CH_arom_), 53.1, 52.8 (2 OCH_3_), 33.0 (S-CH), 32.0 (broad, 2 CH_2_) ppm; HRMS (ESI): [M + Na]^+^ calcd for C_21_H_18_NaO_4_S, 389.08180; found, 389.08178; anal. calcd for C_21_H_18_O_4_S: C, 68.82; H, 4.95; S, 8.75; found: C, 68.71; H, 5.06; S, 8.82.

**Dimethyl 4b*H*-benzo[4,5]cyclohepta[1,2,3-*ij*]isothiochromene-6,7-dicarboxylate (4c):** Yield: 83 mg (46%). Orange solid; mp 139.7–140.0 °C (purified chromatographically); IR (KBr) ν: 3018 (w), 2947 (w), 1728 (s), 1725 (s), 1596 (w), 1563 (w), 1433 (w), 1264 (s),1229 (s), 1097 (w), 771 (w) cm^−1^; ^1^H NMR (600 MHz, CDCl_3_) δ 7.77 (d, *J* = 6 Hz, 1H_arom_), 7.49–7.45 (m, 2H_arom_), 7.42 (d, *J* = 6 Hz, 1H_arom_), 7.36 (d, *J* = 6 Hz, 1H_arom_), 7.30, 7.27 (2d, *J* = 6 Hz, 2 olefinic HC=), 7.23 (d, *J* = 6 Hz, 2H_arom_), 4.49 (s, S-CH), 3.92, 4.00 (2 s, 6H, 2 OCH_3_) ppm; ^13^C NMR (150 MHz, CDCl_3_) δ 167.8, 164.2 (2 C=O), 139.9, 135.8, 134.6, 132.5, 130.3, 128.8, 124.2 (7 C(sp^2^)), 132.2, 130.1, 129.3, 127.8, 127.6, 126.7, 126.7, 126.6, 125.2 (7 CH_arom_ + 2 CH_olefin_), 53.2, 52.8 (2 OCH_3_), 41.8 (S-CH) ppm; HRMS (MALDI–TOF): [M + Na]^+^ calcd for C_21_H_16_NaO_4_S, 387.0668; found, 387.0667.

**Methyl 11,12-dihydro-4b*H*-benzo[4,5]cyclohepta[1,2,3-*ij*]isothiochromene-7-carboxylate (5b):** Yield: 288.5 mg (94%). Yellow solid; mp 116.0–116.5 °C (chromatographic purification); IR (KBr) ν: 3059 (w), 2946 (w), 1702 (s), 1591 (w), 1431 (w), 1221 (s), 1068 (m), 756 (m) cm^−1^; ^1^H NMR (600 MHz, CDCl_3_) δ 7.93–7.87 (m, 1H_arom_), 7.84–7.80 (m, 1H_arom_), 7.61 (br s, S-CH=), 7.28–7.23 (m, 2H_arom_), 7.21–7.17 (m, 2H_arom_), 7.03 (br s, 1H_arom_), 5.79 (br s, S-CH), 3.87 (s, 3H, OCH_3_), 3.55 (br s, 1CH), 3.35 (br s, 1CH), 3.15 (br s, 1CH), 3.03–2.95 (m, 1CH) ppm; ^13^C NMR (150 MHz, CDCl_3_) δ 165.3 (C=O), 142.0, 138.7, 137.6, 135.2, 125.7, 125.4 (6 C(sp^2^)), 132.4, 130.6, 129.3, 128.0, 127.8, 127.1, 126.6, 126.1 (7 CH_arom_ + S-CH=), 51.9 (OCH_3_), 35.0 (S-CH), 32.2 (broad, 2 CH_2_) ppm; HRMS (MALDI–TOF): [M + Na]^+^ calcd for C_19_H_16_NaO_2_S, 331.0761; found, 331.0769.

**Methyl 4b*H*-benzo[4,5]cyclohepta[1,2,3-*ij*]isothiochromene-7-carboxylate (5c):** Yield: 85 mg (61%). Yellow solid; mp 77.5–78.0 °C (chromatographic purification); IR (KBr) ν: 3057 (w), 2948 (w), 1709 (s), 1644 (m), 1589 (w), 1432 (w), 1235 (s), 1066 (m), 726 (m) cm^−1^; ^1^H NMR (600 MHz, CDCl_3_) δ 7.98 (m, 1H_arom_), 7.95 (d, *J* = 6 Hz, 1H_arom_), 7.75 (d, *J* = 12 Hz, 1H_arom_), 7.43–7.41 (m, 1H_arom_), 7.37–7.34 (m, 2H_arom_), 7.27 (s, 1H_arom_), 7.28–7.24 (m, 1H_arom_), 7.22, 7.18 (2 d, *J* = 12 Hz, 2 olefinic HC=), 4.45 (s, S-CH), 3.86 (s, 3H, OCH_3_) ppm; ^13^C NMR (150 MHz, CDCl_3_) δ 165.2 (C=O), 139.7, 134.9, 134.7, 131.6, 131.1, 125.0 (6 C(sp^2^)), 138.7, 131.9, 131.6, 130.7, 130.2, 128.8, 127.9, 127.8, 126.5, 126.1 (7 CH_arom_ + 2 CH_olefin_ + S-CH=), 51.9 (OCH_3_), 41.3 (S-CH) ppm; MS (ESI) *m/z* (%): 207 (100, [M − 74]^+^), 245 (55, [M − 36]^+^), 297 (45, [M − 16]^+^); anal. calcd for C_19_H_14_O_2_S: C, 74.48; H, 4.61; S, 10.46; found: C, 74.53; H, 4.99; S, 10.64.

**General procedure for the oxidation of thiopyran derivatives 4a,b and 5a,b:** A solution of 1 mmol of the corresponding thiopyran and 3 mmol of *m*CPBA (70% purity) in dichloromethane was stirred at room temperature for 3 days. Then the reaction mixture was extracted with aqueous saturated NaHCO_3_ (3 × 10 mL) and distilled water (1 × 10 mL). The organic phase was dried over anhydrous MgSO_4_ and concentrated in vacuo.

**Methyl 11,12-dihydro-4b*H*-benzo[4,5]cyclohepta[1,2,3-*ij*]isothiochromene-7-carboxylate 5,5-dioxide (6d):** Yield: 88 mg (80%). Colorless crystals; mp 187.5–188.0 °C (MeOH); IR (KBr) ν: 3041 (w), 2950 (w), 1717 (s), 1600 (w), 1433 (w), 1307 (s), 1262 (s), 1130 (s), 785 (m) cm^−1^; ^1^H NMR (600 MHz, CDCl_3_) δ 7.60–7.48 (m, 1H_arom_), 7.36 (br s, 1H_arom_ + S-CH=), 7.32–7.28 (m, 2H_arom_), 7.27–7.20 (m, 3H_arom_), 5.72 (br s S-CH), 3.96 (s, 3H, OCH_3_), 3.58 (br s, 1H), 3.10 (br s, 2H), 2.91 (br s, 1H) ppm; ^13^C NMR (150 MHz, CDCl_3_) δ 164.9 (C=O), 134.5, 134.3, 132.2, 131.0, 130.8, 129.2 (6 C(sp^2^)), 134.1, 133.3, 130.0, 129.7, 129.1, 128.0, 127.5, 126.1 (7 CH_arom_ + S-CH=), 62.5 (OCH_3_), 53.4 (S-CH), 37.3, 34.7 (2 broad signals, 2 CH_2_) ppm; HRMS (ESI): [M + Na]^+^ calcd for C_19_H_16_NaO_4_S, 363.06615; found, 363.06614.

## Supporting Information

File 1 Experimental data for selected compounds **4–6**, details of the crystal structure determination, and the original ^1^H and ^13^C NMR spectra for all products. CCDC-1038599 and 1038600 contain the supplementary crystallographic data for this paper. These data can be obtained free of charge from The Cambridge Crystallographic Data Centre via http://www.ccdc.cam.ac.uk/data_request/cif.
