# Supplementary material for: Hetero-Diels–Alder reactions of hetaryl and aryl thioketones with acetylenic dienophiles
Source: Beilstein J Org Chem. 2015 Apr 28;11:576–82. doi: 10.3762/bjoc.11.63 (PMC4464410; doi:10.3762/bjoc.11.63)
Supplement: File 1 — Experimental data for selected compounds 4–6, details of the crystal structure determination, and the original 1H and 13C NMR spectra for all products. CCDC-1038599 and 1038600 contain the supplementary crystallographic data for this paper. These data can be obtained free of charge from The Cambridge Crystallographic Data Centre via http://www.ccdc.cam.ac.uk/data_request/cif. [file Beilstein_J_Org_Chem-11-576-s001.pdf]

Supporting Information

for

Hetero-Diels–Alder reactions of hetaryl and aryl thioketones with acetylenic dienophiles

Grzegorz Mlostoń[1,*], Paulina Grzelak[1], Maciej Mikina[2], Anthony Linden[3] and Heinz Heimgartner[3,*]

Address: [1]Department of Organic and Applied Chemistry, University of Łódź, Tamka 12, PL 91-403 Łódź, Poland, [2]Center of Molecular and Macromolecular Studies PAS, Sienkiewicza 112, PL 90-363 Łódź, Poland and [3]Department of Chemistry, University of Zürich, Winterthurerstrasse 190, CH-8057 Zürich, Switzerland

Email: Grzegorz Mlostoń - gmloston@uni.lodz.pl; Heinz Heimgartner - heinz.heimgartner@chem.uzh.ch

*Corresponding author

Experimental data for selected compounds 4–6, details of the crystal structure determinations, and the original ^{1}H and ^{13}C NMR spectra for all products

1. Experimental data for selected compounds 4, 5, and 6

*Dimethyl 1*H*-2-benzothiopyran-3,4-dicarboxylate* (**4a**): Yield: 285.6 mg (84%). Colorles crystals; m.p. 91.0–91.5 ^{o}C (MeOH) (lit. [S1] m.p. 90.0-91.0 ^{o}C)

*Dimethyl 7-(*N*-methylpyrrol-2-yl)-*N*-methylpyrrolo[2,3-*c*]thiopyran-4,5-dicarboxylate* (**4d**): Yield: 60.5 mg (35%). Orange solid; m.p. 141.5–142.0 °C (purified chromatographically).

IR (KBr): ν = 311 (w), 2951 (w), 1730 (s), 1729 (s), 1695 (s), 1572 (w), 1433 (m), 1271 (s), 1251 (s),1224 (s), 1199 (m), 1164 (w), 726 (m) cm^{-1}. ^{1}H NMR (600 MHz, $CDCl_3$): δ = 6.61-6.58 (m, 2 H_{arom}), 6.21 (d, *J* = 6Hz, 1 H_{arom}), 5.95-5.93 (m, 1 H_{arom}), 5.45-5.43 (m, 1 H_{arom}), 5.37 (s, 1 S-CH); 3.92 (d, *J* = 6Hz, 3 H, CH_3), 3.91 (s, 3H, CH_3), 3.74, 3.69 (2 s, 6H, 2 OCH_3) ppm. ^{13}C NMR (150 MHz, $CDCl_3$): δ = 164.6, 160.2 (2 C=O), 137.0, 136.1, 134.1, 118.0, 112.5 (5 $C(sp^2)$), 124.1, 123.5, 110.3, 107.3, 106.3 (5 CH_{arom}), 52.5, 52.4 (2 OCH_3), 34.1 (CH_3), 33.5(S-CH), 33.4 (CH_3) ppm. MS (ESI): *m/z* (%) = 345 (100, $[M–1]^+$), 369 (65) $[M+Na]^+$. $C_{17}H_{18}N_2O_4S$ (346.43): calcd. C 58.93, H 5.25, N 8.09, S 9.25; found C 59.33, H 5.25, N 8.24, S 9.21.

*Dimethyl 7-(2-thienyl)-7*H-*thieno[2,3-*c*]thiopyran-4,5-dicarboxylate* (**4e**): Yield: 123.0 mg (35%). Yellowish crystals, m.p. 94–96 °C (lit. [S2], m.p. 94–95 °C).

*Methyl 1-phenyl-1*H-*2-benzothiopyran-4-carboxylate* (**5a**) [S3]: Yield: 191.8 mg (68%). Yellow oil. IR (film): ν = 3059 (w), 2949 (w), 1716 (s), 1578 (w), 1490 (w), 1436 (m), 1239 (s), 1031 (m), 745 (m) cm^{-1} . ^{1}H NMR (600 MHz, $CDCl_3$): δ = 8.10 (d, *J* = 7.74 Hz, 1 H_{arom}), 7.76 (s, S-CH=), 7.44 (t, *J* = 7.56 Hz, 1 H_{arom}), 7.38-7.25 (m, 6 H_{arom}), 7.05 (d, *J* = 7.62 Hz, 1 H_{arom}), 5.21 (s, S-CH), 3.90 (s, 3 H, OCH_3) ppm. ^{13}C NMR (150 MHz, $CDCl_3$): δ = 165.0 (C=O), 139.9, 131.1, 130.8, 126.9 (4 $C(sp^2)$), 135.4, 128.8, 128.6, 128.2, 127.8, 127.7, 127.2, 127.1 (7 CH_{arom} + S-CH=); 51.9 (OCH_3); 47.1 (S-CH) ppm. MS (ESI): *m/z* (%) = 281 (100, $[M–1]^+$), 282 (23, $[M]^+$) , 283 (48, $[M+1]^+$).

*Methyl 7-(2-thienyl)-7*H-*thieno[2,3-*c*]thiopyran-4-carboxylate* (**5d**): Yield: 244 mg (83%). Green colored solid; m.p. 85.0-85.5 °C (chromatographic purification). IR (KBr): ν = 3038 (w), 2948 (w), 1701 (s), 1544 (w), 1437 (m), 1255 (s), 1068 (w), 716 (m) cm^{-1}. ^{1}H NMR (600 MHz, $CDCl_3$): δ = 7.74 (*d*, *J* = 6 Hz, 1 H_{arom}), 7.59 (s, S-CH=), 7.26-7.21 (m, 2 H_{arom}), 6.92-6.90 (m, 1 H_{arom}), 6.88-6.87 (m, 1 H_{arom}), 5.76 (s, S-CH), 3.86 (s, 3 H, OCH_3) ppm. ^{13}C NMR (150 MHz, $CDCl_3$): δ = 164.3 (C=O), 144.5, 131.8, 130.7, 123.1 (4 $C(sp^2)$), 131.7, 127.4, 126.9, 126.2, 126.0, 122.9 (5 CH_{arom}+ S-CH=), 51.9 (OCH_3), 38.5 (S-CH) ppm. HRMS (MALDI TOF MS): calcd. for $C_{13}H_{10}NaO_2S_3$ $[M+Na]^+$ 316.9742; found 316.9741.

*Methyl 7-(selenophen-2-yl)-7*H*-selenopheno[2,3-*c*]thiopyran-4-carboxylate* (**5e**) and *methyl 7-(2-furanyl)-7*H*-thiopyrano[3,4-*b*]furan-4-carboxylate* (**5f**) were identified only in the crude mixtures and could not be isolated in pure form. Yields of **5e** and **5f** were calculated based on the ^{1}H NMR spectra of crude mixtures recorded using a weighted standard (1,1,2,2-tetrachloroethane).

*Methyl 7-phenyl-7*H*-thieno[2,3-*c*]thiopyran-4-carboxylate* (**5g**): Yield: 213 mg (74%). Green solid; m.p. 88.5-89.0 °C (chromatographic purification). IR (KBr): ν = 3037 (w), 2948 (w), 1701 (s), 1544 (w), 1436 (m), 1254 (s), 1068 (w), 716 (m) cm^{-1}. ^{1}H NMR (600 MHz, $CDCl_3$): δ = 7.78 (d, *J* = 5.3 Hz, 1 H_{arom}), 7.61 (s, S-CH=), 7.27 (dd, *J* = 1.2, 5.1 Hz, 2 H_{arom}), 7.25 (d, *J* = 5.3 Hz, 1 $H_{arom,}$), 6.94-6.92 (m, 1 H_{arom}), 6.91-6.88 (m, 2H_{arom}), 5.78 (s, S-CH), 3.87 (s, 3 H, OCH_3) ppm. ^{13}C NMR (150 MHz, $CDCl_3$): δ = 164.3 (C=O), 144.5, 130.7, 127.4, 126.0 (4 C(sp^2)), 131.8, 126.9, 126.2, 123.1, 122.9 (5 CH_{arom} + S-CH=), 52.0 (OCH_3), 38.5 (S-CH) ppm. HRMS (MALDI TOF): calcd. for $C_{15}H_{12}NaO_2S_2$ $[M+Na]^+$ 311.0180; found 311.0176.

*Dimethyl 1*H*-2-benzothiopyran-3,4-dicarboxylate S,S-dioxide* (**6a**) [S4]: Yield: 336 mg (90%). Colorless crystals; m.p. 188.5-188.8 °C (MeOH). IR (KBr): ν = 3065 (w), 2926 (w), 1737 (s), 1716 (s), 1602 (m), 1559 (w), 1438 (w), 1325 (s), 1248 (s), 1227 (s), 1132 (s), 1073 (w), 762 (m) cm^{-1}. ^{1}H NMR (600 MHz, $CDCl_3$): δ = 7.54-7.49 (m, 2 H_{arom}), 7.48-7.43 (m, 4 H_{arom}), 7.42-7.39 (m, 2 H_{arom}), 7.25 (*d*, *J* = 6.95 Hz, 1 H_{arom}), 5.48 (s, S-CH), 4.05, 3.93 (2 s, 6 H, 2 OCH_3) ppm. ^{13}C NMR (150 MHz, $CDCl_3$): δ = 165.3, 161.0 (2 C=O), 146.0, 133.8 130.4, 129.9, 126.9 (5 C(sp^2)), 134.8, 132.6, 130.8, 129.8, 129.5, 129.1, 128.9 (7 CH_{arom}), 70.0 (broad, for 2 OCH_3), 53.5 (S-CH) ppm. MS (ESI): *m/z* (%) = 373 (20, $[M+1]^+$), 395 (100, $[M+Na]^+$). $C_{19}H_{16}O_6S$ (372.41): calcd. C 61.29, H 4.30, S 8.60; found C 61.34, H 4.27, S 8.91.

*Methyl 1*H*-2-benzothiopyran-4-dicarboxylate S,S-dioxide* (**6b**): Yield: 104 mg (94%). White solid; m.p. 137.5 – 138.0 °C (isolated crude product). IR (KBr): ν = 3061 (w), 2953 (w), 1733 (s), 1602 (w), 1447 (w), 1316 (s), 1236 (s), 1125 (s), 1020 (m), 776 (m) cm^{-1}. ^{1}H NMR (600 MHz, $CDCl_3$): δ = 7.82 (d, *J* = 8.0 Hz, 1 H_{arom}), 7.50 (t, *J* = 7.6 Hz, 1 H_{arom}), 7.45-7.40 (m, 4 H_{arom}), 7.37-7.34 (m, 2 H_{arom}), 7.22 (d, *J* = 7.6 Hz, 1 H_{arom}), 7.15

(s, HC=), 5.42 (s, 1 CH), 3.98 (s, 3 H, OCH_3) ppm. ^{13}C NMR (150 MHz, $CDCl_3$): δ = 164.6 (C=O), 139.6, 133.6, 128.8 (4 C(sp^2)), 131.1, 130.6 130.4, 130.2, 129.4, 129.0, 128.9, 127.0 (9 CH_{arom} + S-CH=), 68.9 (OCH_3), 53.3 (S-CH) ppm. MS (ESI): *m/z* (%) = 304 (70, $[M–10]^+$), 314 (20, $[M]^+$), 337 (100, $[M+Na]^+$). $C_{17}H_{14}O_4S$ (314.36): calcd. C 64.95, H 4.49, S 10.20; found C 65.06, H 4.56, S 10.15.

Dimethyl 11,12-dihydro-4bH-benzo[4,5]cyclohepta[1,2,3-ij]isothiochromene-6,7-dicarboxylate 5,5-dioxide (**6c**): Yield: 76 mg (74%). White solid; m.p. 207.5-208.0 °C (isolated crude product). IR (KBr): ν = 3014 (w), 2953 (w), 1729 (s), 1717 (s), 1597 (w), 1569 (m), 1439 (m), 1322 (s), 1262 (s), 1231 (s), 1147 (s), 1102 (w), 759 (m) cm^{-1}. ^1H NMR (600 MHz, $CDCl_3$): δ = 7.44-7.38 (m, 1 H_{arom}), 7.35-7.31 (m, 3 H_{arom}), 7.24-7.14 (m, 3 H_{arom}), 5.75 (br. s S-CH), 4.00, 3.97 (2 s, 6 H, 2 OCH_3), 3.59 (br. s, 1 H), 3.13 (br. s, 2 H), 2.92 (br. s, 1 H) ppm. ^{13}C NMR (150 MHz, $CDCl_3$): δ = 169.9, 165.4 (2 C=O), 134.6, 132.7, 132.3, 130.6, 130.5, 129.2, 126.2 (7 C(sp^2)), 133.7, 131.0, 130.2, 129.8, 128.2, 127.9, 126.6 (7 CH_{arom}), 65.8, 62.5 (2 OCH_3), 53.5 (S-CH), 37.2, 34.6 (2 broad signals, 2 CH_2) ppm. HRMS (MALDI TOF): calcd. for $C_{21}H_{18}NaO_6S$ $[M+Na]^+$ 421.0723; found 421.0722.

2. X-ray crystallography

Crystal data for **4b**: $C_{21}H_{18}O_4S$, M_r = 366.43, colorless, prism, 0.15 × 0.15 × 0.22 mm, T = 160(1) K, monoclinic, *I*2/*a*, Z = 8, a = 21.68846(14), b = 10.06163(5), c = 16.63858(12) Å, β = 109.0246(8)°, V = 3432.57(4) Å^3, D_x = 1.418 g cm^{-3}, μ(Cu $K\alpha$) = 1.885 mm^{-1}, ω scans, $2\theta_{(max)}$ = 148.5°, transmission factors (min; max) = 0.545; 1.000, 31829 reflections measured, 3481 symmetry independent reflections, 3435 reflections with $I > 2\sigma(I)$, 3481 reflections used in refinement, 238 parameters, $R(F)$ [$I > 2\sigma(I)$ reflections] = 0.0285, $wR(F^2)$ (all data) = 0.0748, $w = [\sigma^2(F_o^2) + (0.0357P)^2 + 2.8020P]^{-1}$ where $P = (F_o^2 + 2F_c^2)/3$, goodness of fit = 1.055, secondary extinction coefficient = 0.00042(6), final Δ_{max}/σ = 0.001, $\Delta\rho$(max; min) = 0.28; –0.22 e Å^{-3}. Crystals from methanol.

Crystal data for **6d**: $C_{19}H_{16}O_4S$, M_r = 340.39, colorless, prism, 0.15 × 0.17 × 0.24 mm, T = 160(1) K, triclinic, *P*−1, Z = 4, a = 9.2396(2), b = 12.3639(3), c = 14.1109(4) Å, α = 89.197(2), β = 73.619(2)°, γ = 88.599(2)°, V = 1546.05(7) Å^3, D_x = 1.462 g cm^{-3}, μ(Mo $K\alpha$) = 0.230 mm^{-1}, ω scans, $2\theta_{(max)}$ = 56.6°, transmission factors (min; max) = 0.909;

1.000, 40958 reflections measured, 12307 symmetry independent reflections, 8546 reflections with $I > 2\sigma(I)$, 12307 reflections used in refinement, 436 parameters, $R(F)$ [$I > 2\sigma(I)$ reflections] = 0.0371, $wR(F^2)$ (all data) = 0.1041, $w = [\sigma^2(F_o^2) + (0.0568P)^2]^{-1}$ where $P = (F_o^2 + 2F_c^2)/3$, goodness of fit = 1.042, final Δ_{max}/σ = 0.001, $\Delta\rho$(max; min) = 0.37; −0.48 e Å^{-3}. Crystals from CH_2Cl_2.

All measurements were made on an *Agilent Technologies SuperNova* area-detector diffractometer [S5] using Cu Kα radiation (λ = 1.54184 Å) in the case of **4b** and Mo Kα radiation (λ = 0.71073 Å) in the case of **6d** from a micro-focus X-ray source and an *Oxford Instruments Cryojet XL* cooler. Data reduction was performed with *CrysAlisPro* [S5]. The intensities were corrected for *Lorentz* and polarization effects, and an empirical absorption correction using spherical harmonics [S5] was applied. The space groups of **4b** and **6d** were determined by the systematic absences, packing considerations, a statistical analysis of intensity distribution, and the successful solution and refinement of the structure. The chosen crystal of **6d** was a non-merohedral twin resulting from a rotation of 180° about [0 1 0] with a twin matrix of [–1 0 0 / 0 1 0 / 0 0 –1] and the major twin fraction in the selected crystal is 0.8861(4). All reflections from both twin components were integrated; a total of 11129 and 11104 non-overlapping reflections from twin components 1 and 2, respectively, plus 18725 reflections from both twin components that were overlapping by more than 80%. Equivalent reflections were merged. The data collection and refinement parameters are given above, and views of the molecules are shown in the *Figures*. The structures were solved by direct methods using *SHELXS-2013* [S6], which revealed the positions of all non-H-atoms. In the case of **6d**, there are two symmetry-independent molecules in the asymmetric unit. The atomic coordinates of the two molecules were tested carefully for a relationship from a higher symmetry space group using the program *PLATON* [S7], but none could be found. The non-H-atoms of **4b** and **6d** were refined anisotropically. All of the H-atoms were placed in geometrically calculated positions and refined by using a riding model where each H-atom was assigned a fixed isotropic displacement parameter with a value equal to 1.2Ueq of its parent C-atom (1.5Ueq for the methyl groups). The refinement of each structure was carried out on F^2 by using full-matrix least-squares procedures, which minimized the function $\Sigma w(F_o^2 - F_c^2)^2$. A correction for secondary extinction was applied for **4b**. Neutral atom scattering factors for non-H-atoms were taken from ref.

[S8], and the scattering factors for H-atoms were taken from ref. [S9]. Anomalous dispersion effects were included in F_c; [S10] the values for *f′* and *f″* were those of ref. [S11]. The values of the mass attenuation coefficients are those of ref. [S12]. The *SHELXL-2014* program [S13] was used for all calculations. CCDC-1038599 and 1038600 contain the supplementary crystallographic data for this paper. These data can be obtained free of charge from The *Cambridge Crystallographic Data Centre*, *via* www.ccdc.cam.ac.uk/data_request/cif.

References

[S1] Gotthardt, H.; Nieberl, S. *Liebigs Ann. Chem.* **1980**, 867–872. doi: 10.1002/jlac.198019800607.

[S2] Mloston, G.; Urbaniak, K.; Gebicki, K.; Grzelak, P.; Heimgartner, H. *Heteroatom Chem.* **2014**, *25*, 548–555. doi: 10.1002/hc.21191.

[S3] Rapp, J.; Huisgen, R. *Tetrahedron* **1997**, *53*, 961–970. doi: 10.1002/chin.199716171.

[S4] Okuma, K.; Kojima, K.; Koga, Y.; Shioji, K. *Heterocycles* **2000**, *52*, 1021–1024. doi: 10.3987/COM-99-S118.

[S5] *CrysAlisPro*, Version 1.171.37.31d and 1.171.37.35, Agilent Technologies, Yarnton, Oxfordshire, England, 2014.

[S6] Sheldrick, G. M. *Acta Crystallogr. Sect. A*, **2008**, *64*, 112–122. doi: 10.1107/S0108767307043930.

[S7] Spek, A. L. *Acta Crystallog*r. *Sect. D*, *C*, **2015**, *71*, 9–18. doi: 10.1107/S2053229614024929.

[S8] Maslen, E. N.; Fox, A. G.; O'Keefe, M. A. in 'International Tables for Crystallography', Ed. Wilson, A. J. C. Kluwer Academic Publishers, Dordrecht, **1992**, Vol. C, Table 6.1.1.1, pp. 477–486.

[S9] Stewart, R. F.; Davidson, E. R.; Simpson, W. T. *J. Chem. Phys.* **1965**, *42*, 3175–3187. doi: 10.1063/1.1696397.

[S10] Ibers, J. A.; Hamilton, W. C. *Acta Crystallogr.* **1964**, *17*, 781–782. doi: 10.1107/S0365110X64002067.

[S11] Creagh, D. C.; McAuley, W. J. in 'International Tables for Crystallography', Ed. Wilson, A. J. C. Kluwer Academic Publishers, Dordrecht, **1992**, Vol. C, Table 4.2.6.8, pp. 219–222.

[S12] Creagh, D. C.; Hubbell, J. H. in 'International Tables for Crystallography', Ed. Wilson, A. J. C. Kluwer Academic Publishers, Dordrecht, **1992**, Vol. C, Table 4.2.4.3, pp. 200–206.

[S13] Sheldrick, G. M. Acta Crystallogr. Sect. C, 2015, 71, 3–8. doi: 10.1107/S2053229614024218.

*2. Collection of the ^{1}H- and ^{13}C-NMR spectra for the described compounds **4**, **5**, and **6***

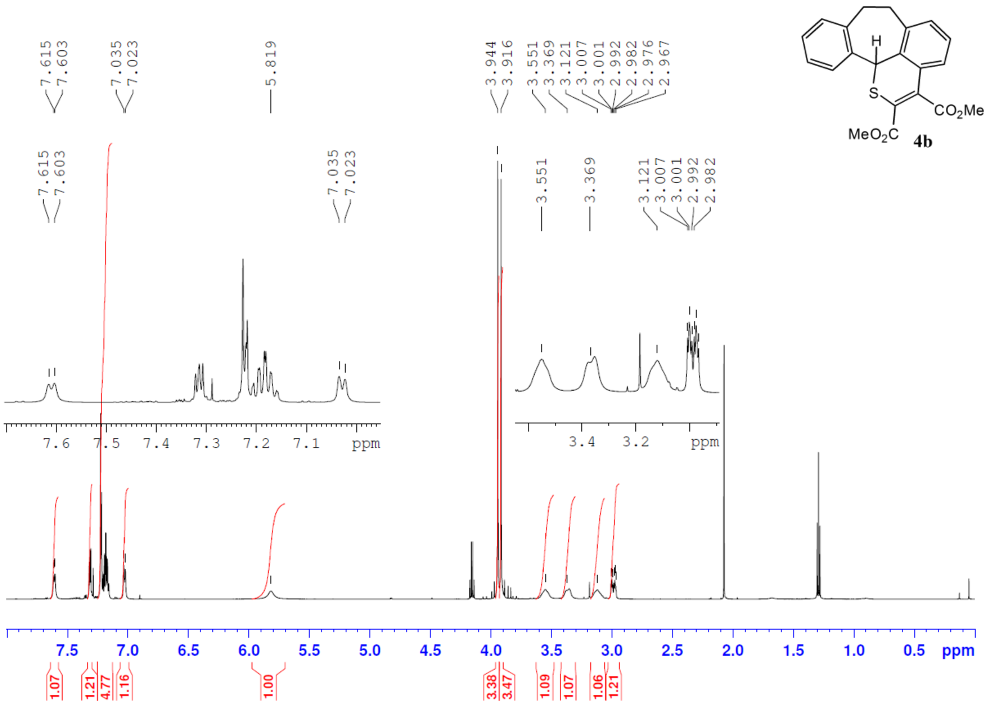

Figure S1: The ^{1}H NMR spectrum of compound **4b**.

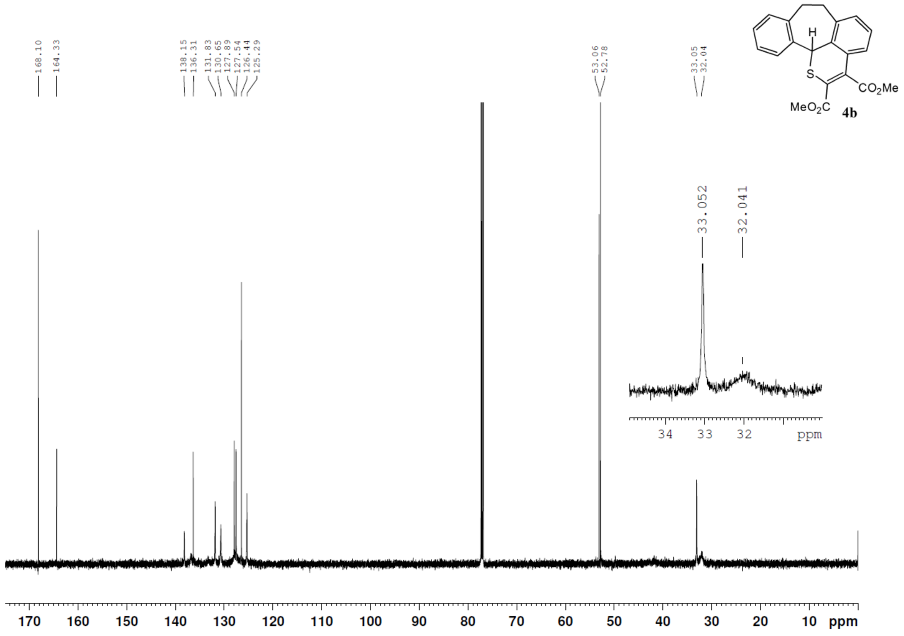

Figure S2: The ^{13}H NMR spectrum of compound **4b**.

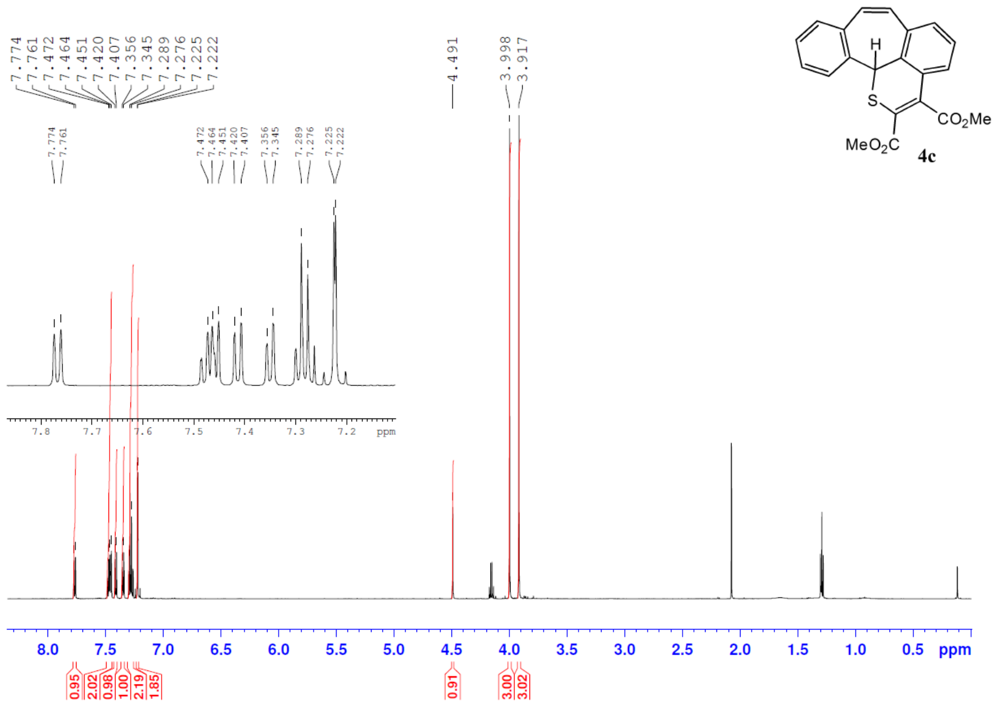

Figure S3: The ^{1}H NMR spectrum of compound **4c**.

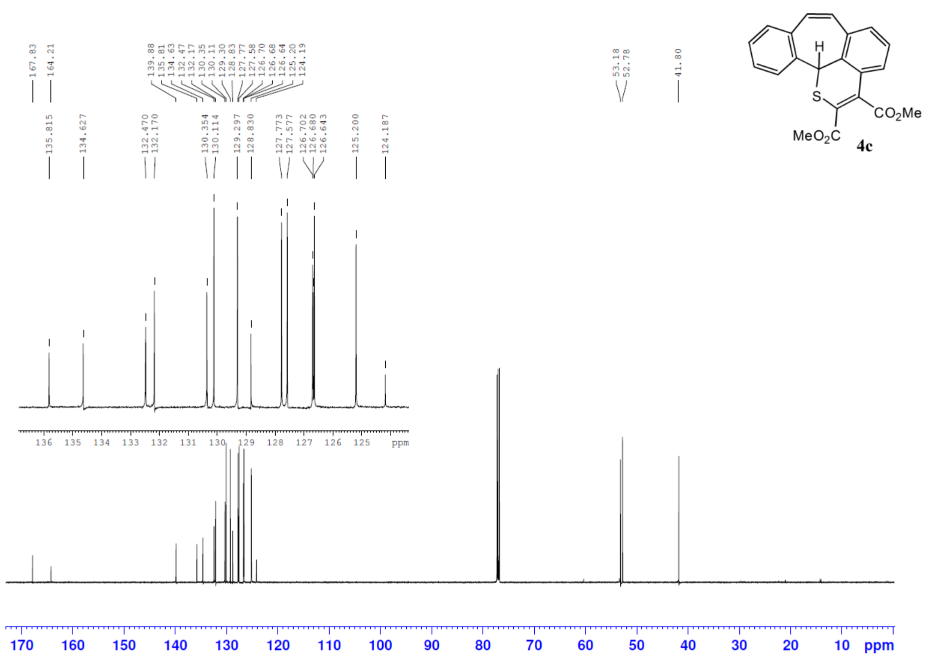

Figure S4: The ^{13}C NMR spectrum of compound **4c**.

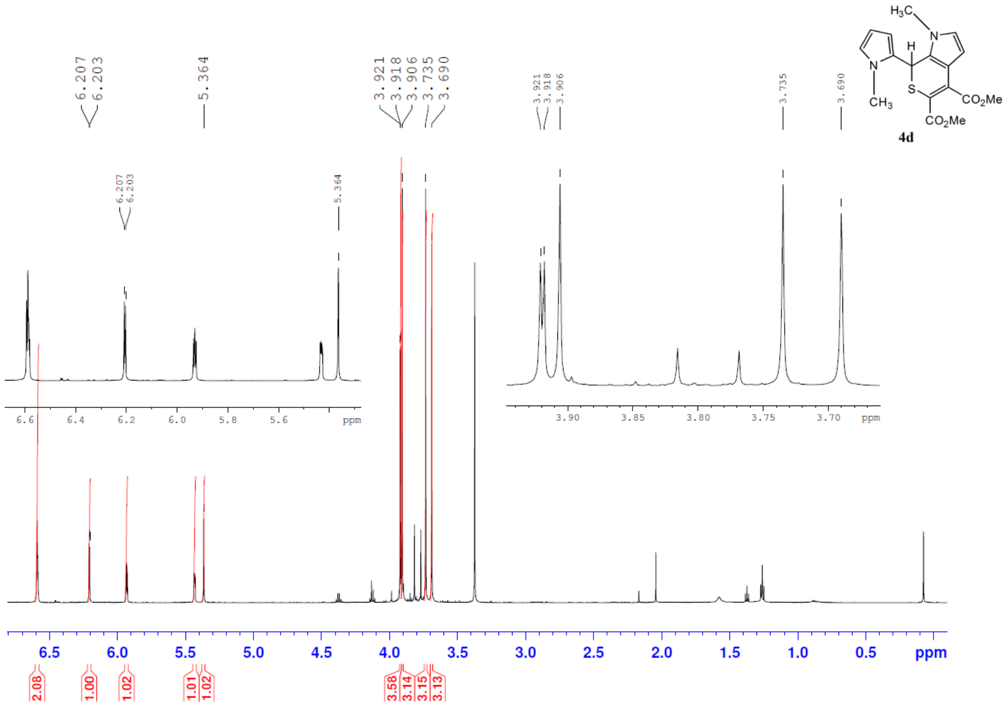

Figure S5: The ^{1}H NMR spectrum of compound **4d**.

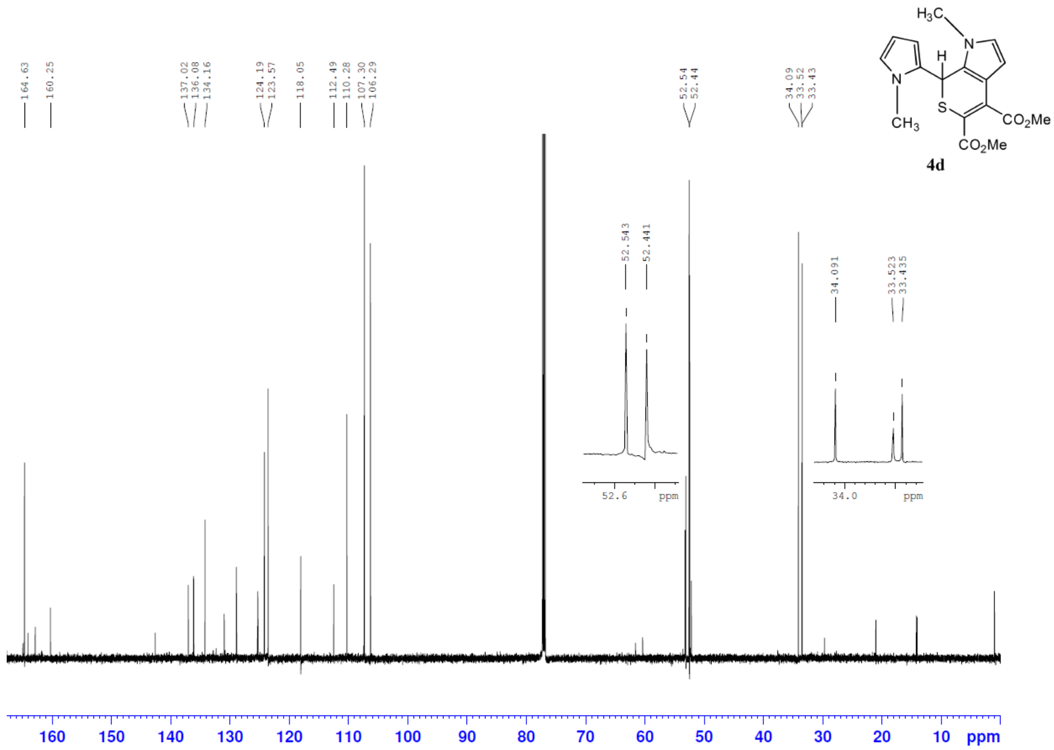

Figure S6: The ^{13}C NMR spectrum of compound **4d**.

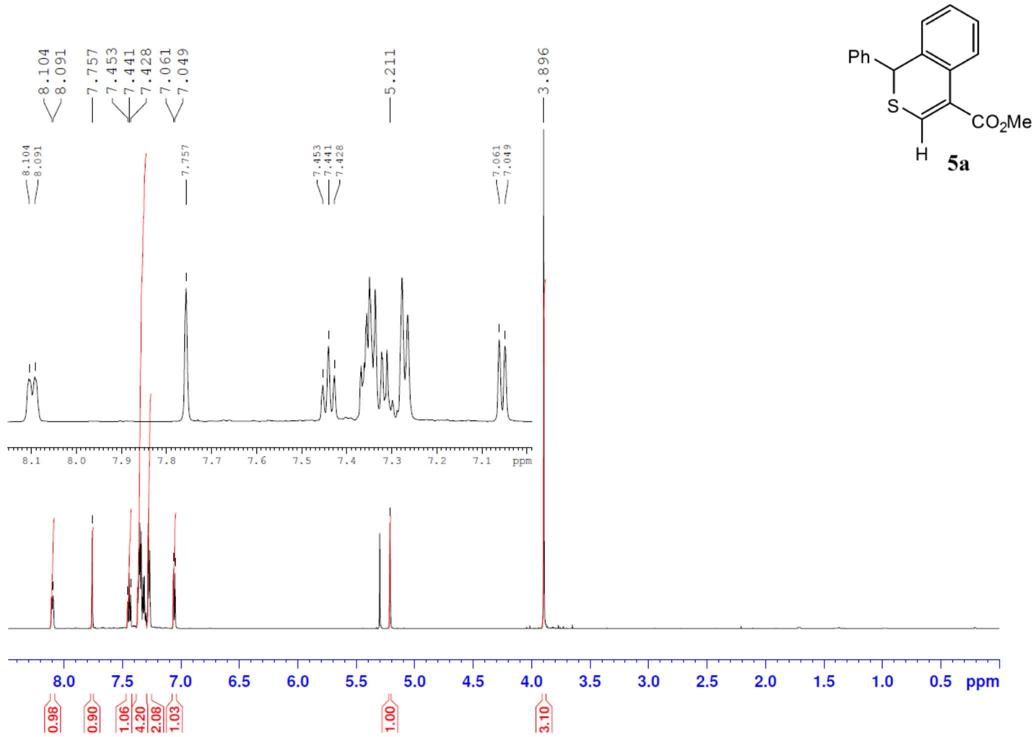

Figure S7: The ^{1}H NMR spectrum of compound **5a**.

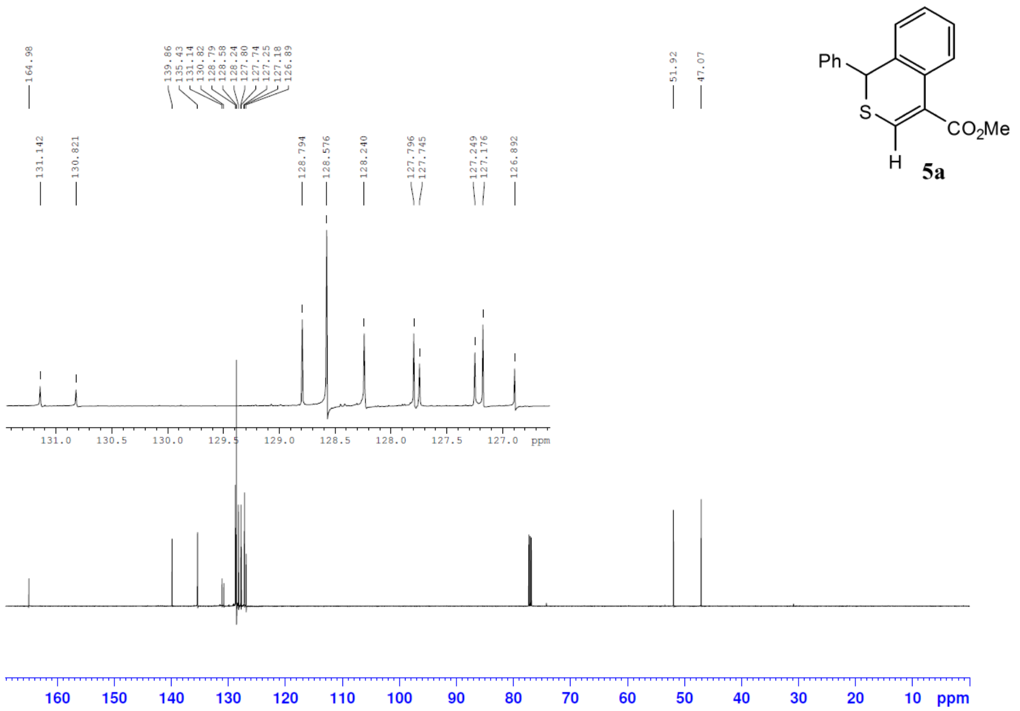

Figure S8: The ^{13}C NMR spectrum of compound **5a**.

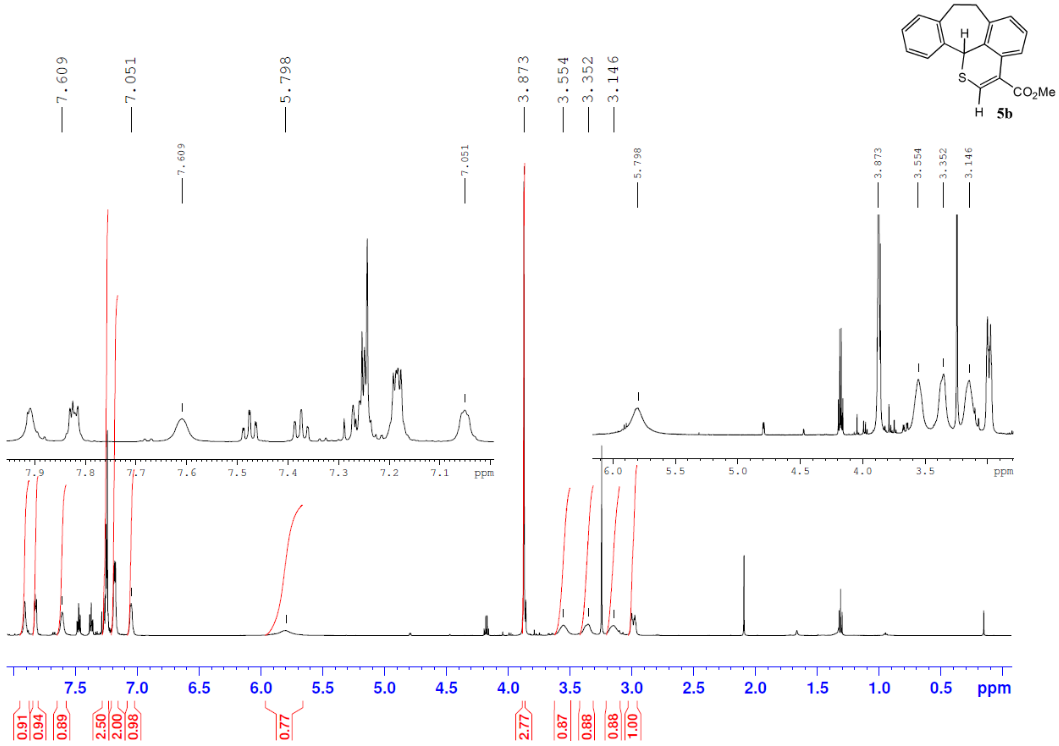

Figure S9: The ^{1}H NMR spectrum of compound **5b**.

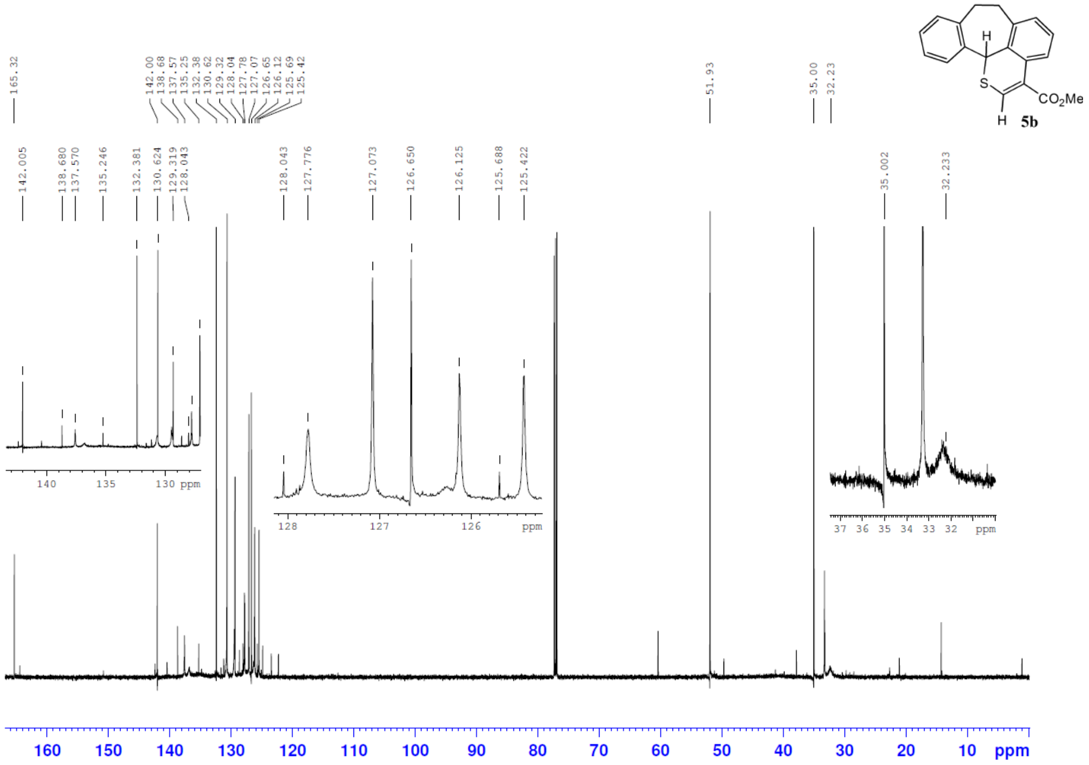

Figure S10. The ^{13}C NMR spectrum of compound **5b**.

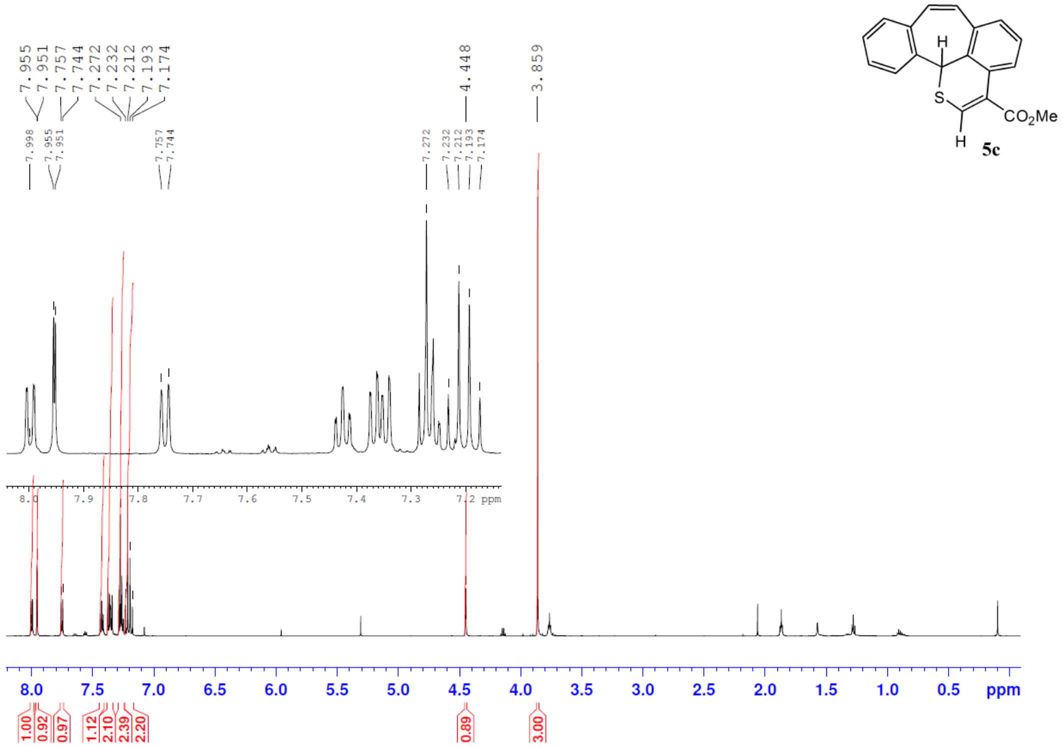

Figure S11: The ^{1}H NMR spectrum of compound **5c**.

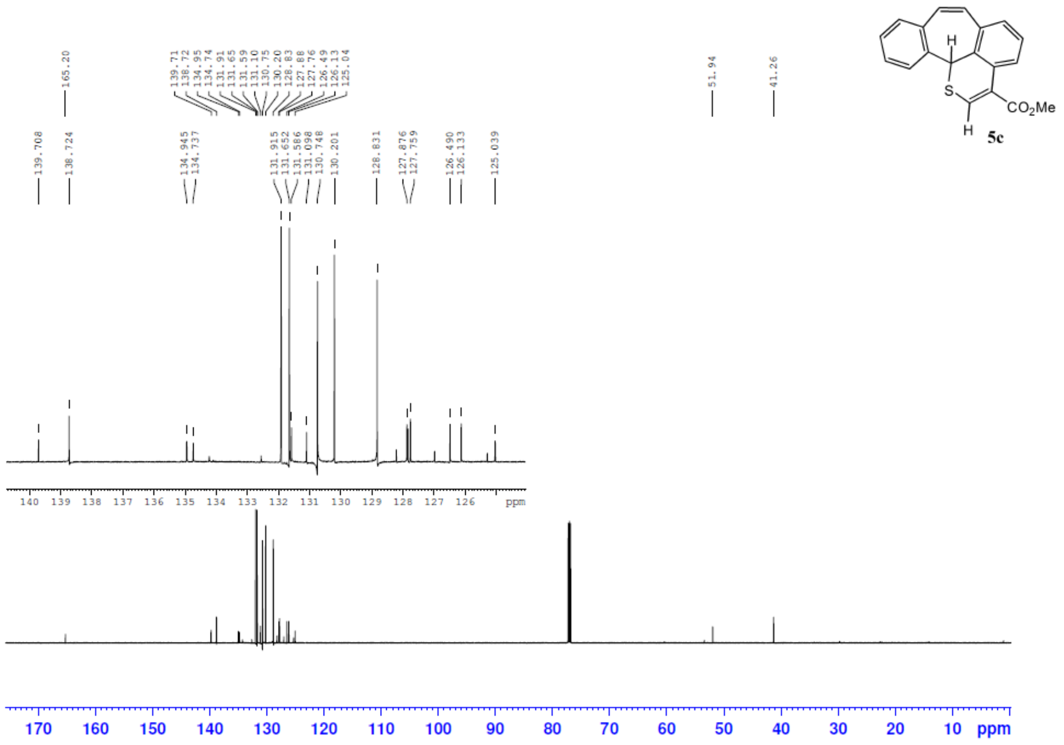

Figure S12: The ^{13}C NMR spectrum of compound **5c**.

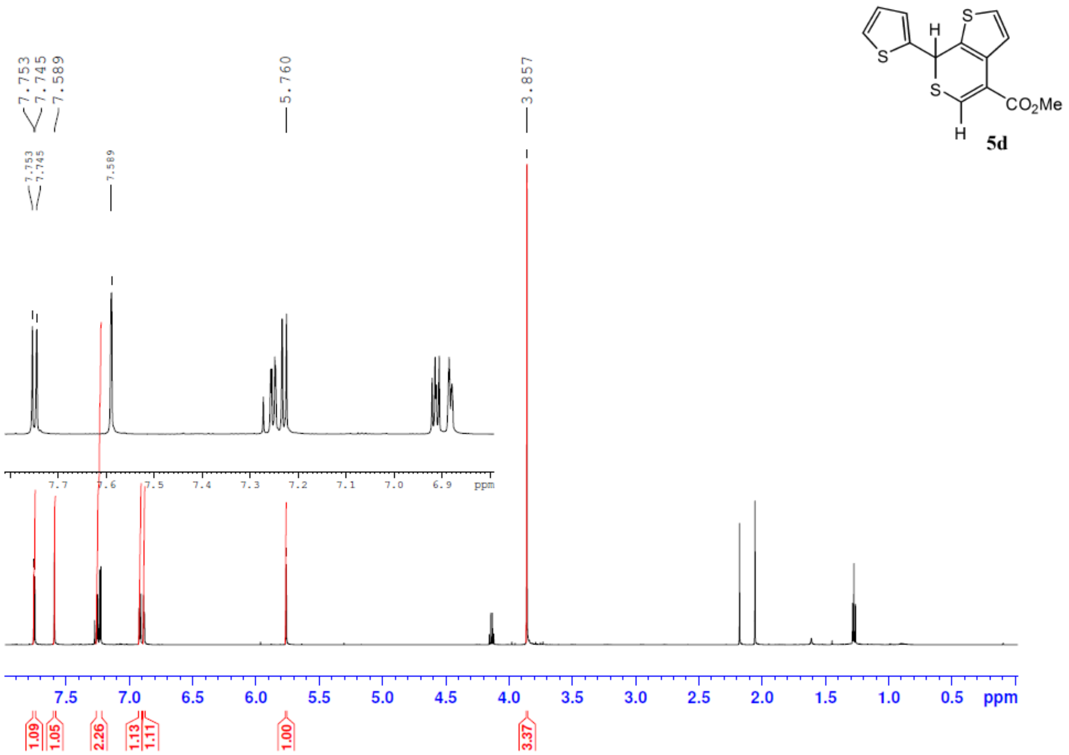

Figure S13: The ^{1}H NMR spectrum of compound **5d**.

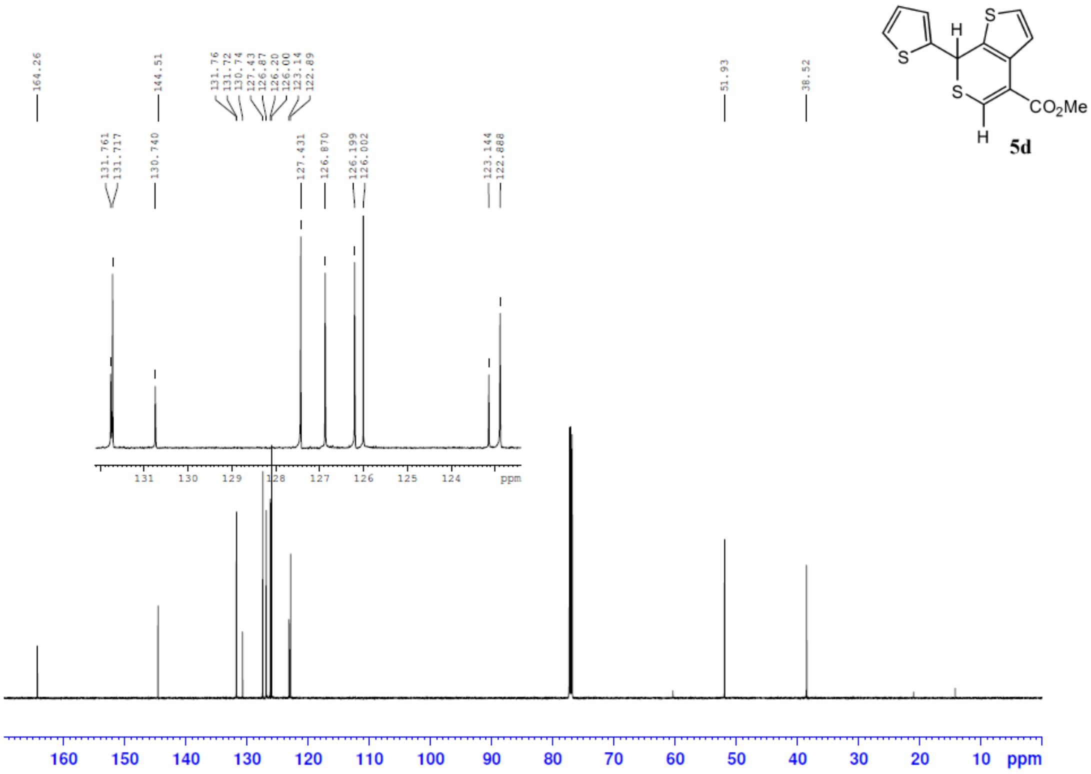

Figure S14: The ^{13}C NMR spectrum of compound **5d**.

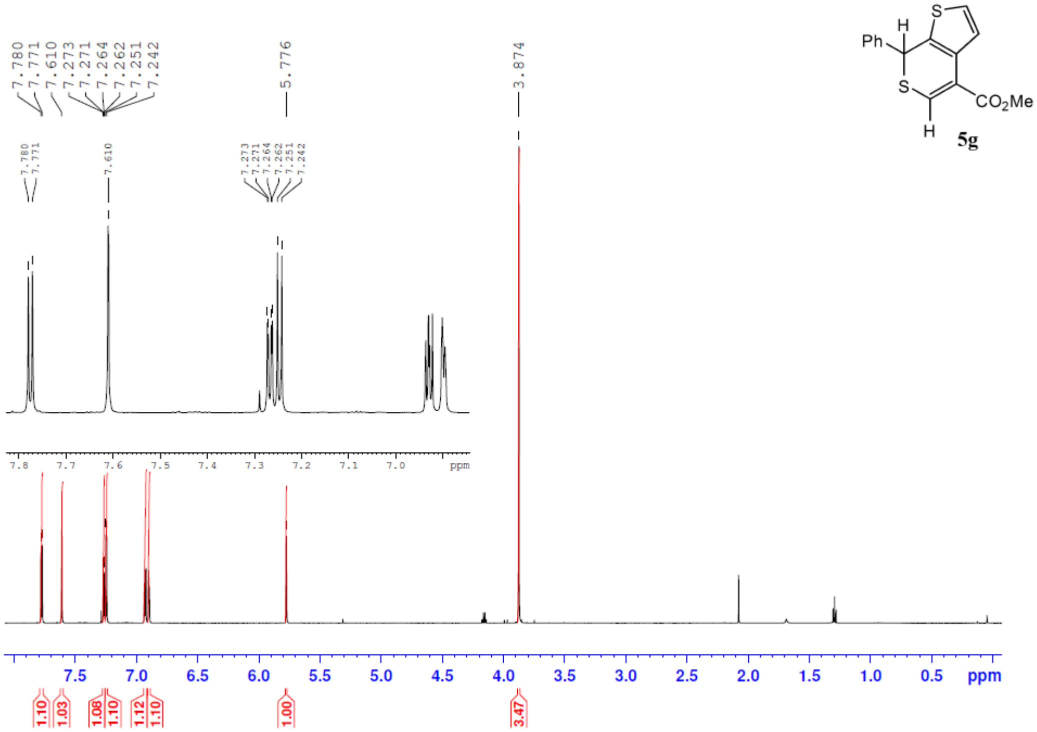

Figure S15: The ^{1}H NMR spectrum of compound **5g**.

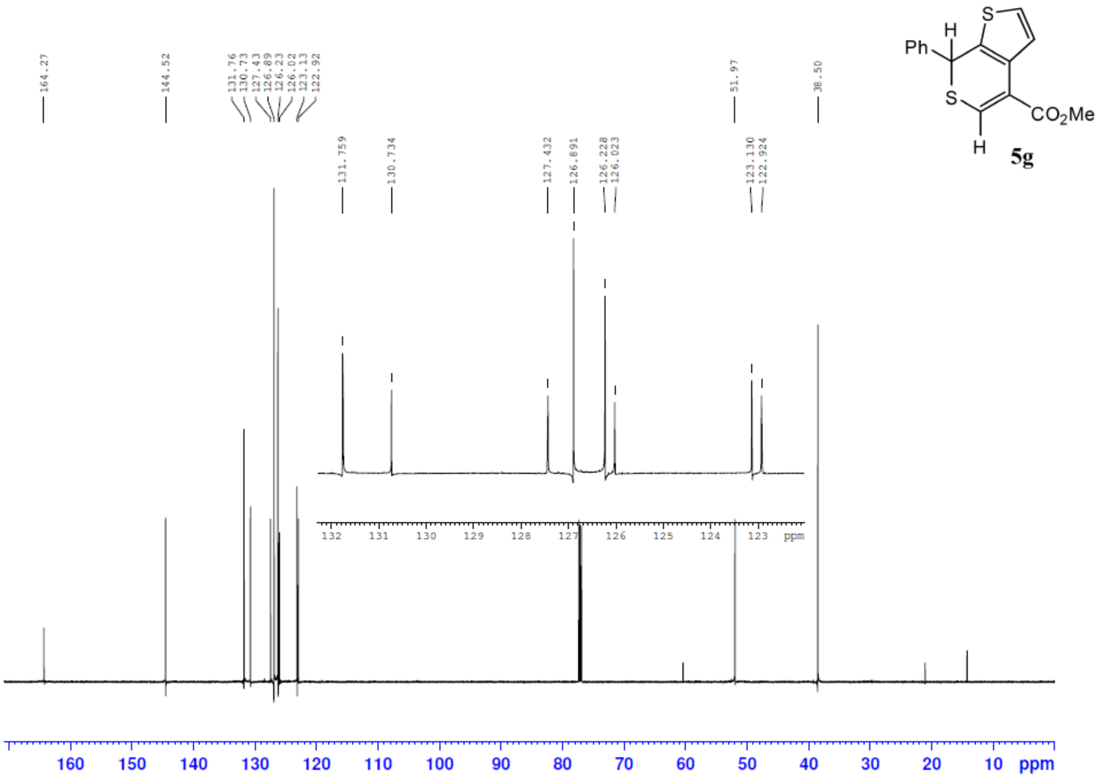

Figure S16: The ^{13}C NMR spectrum of compound **5g**.

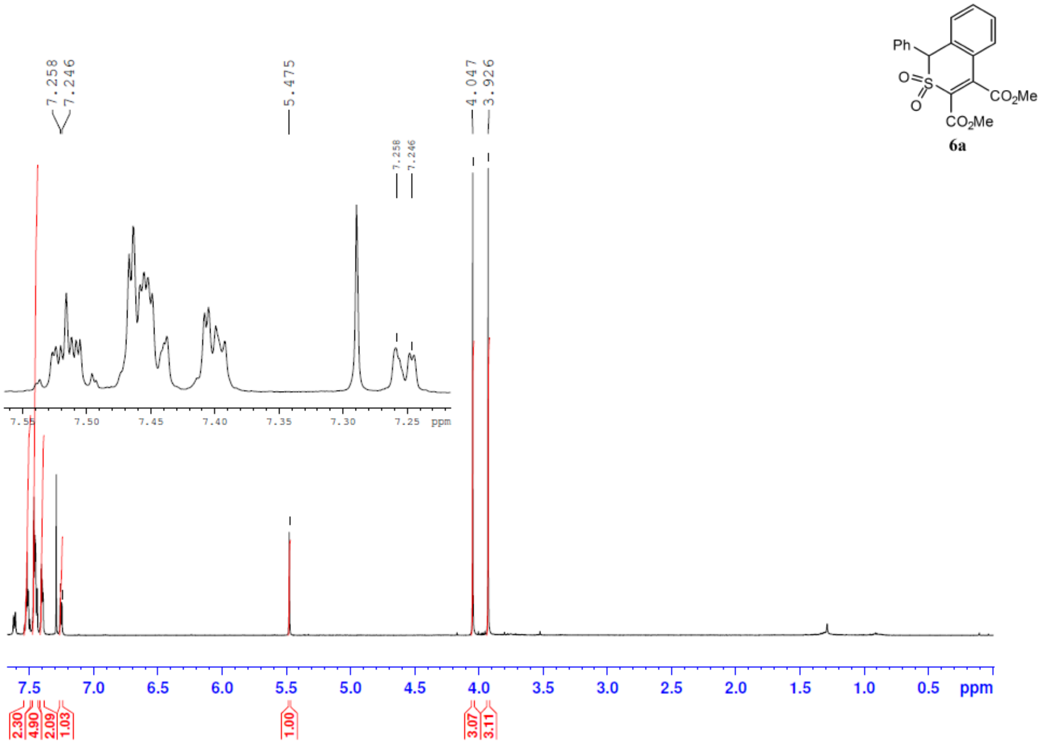

Figure S17: The ^{1}H NMR spectrum of compound **6a**.

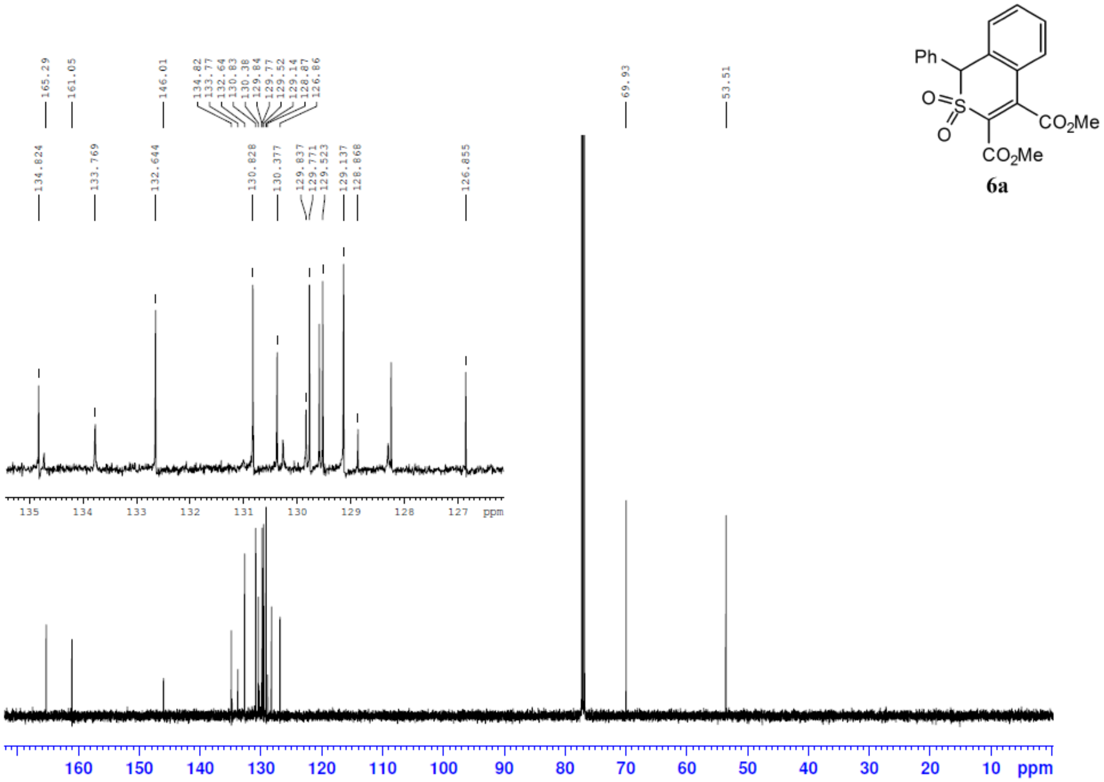

6a

Figure S18:. The ^{13}C NMR spectrum of compound **6a**.

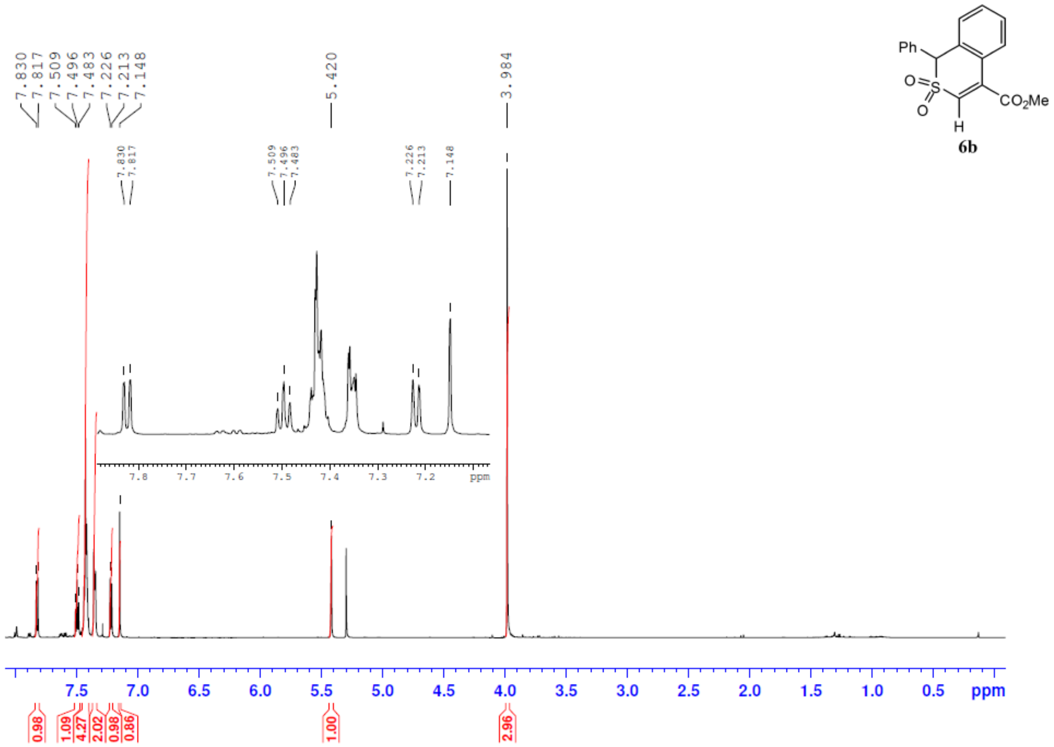

6b

Figure S19: The ^{1}H NMR spectrum of compound **6b**.

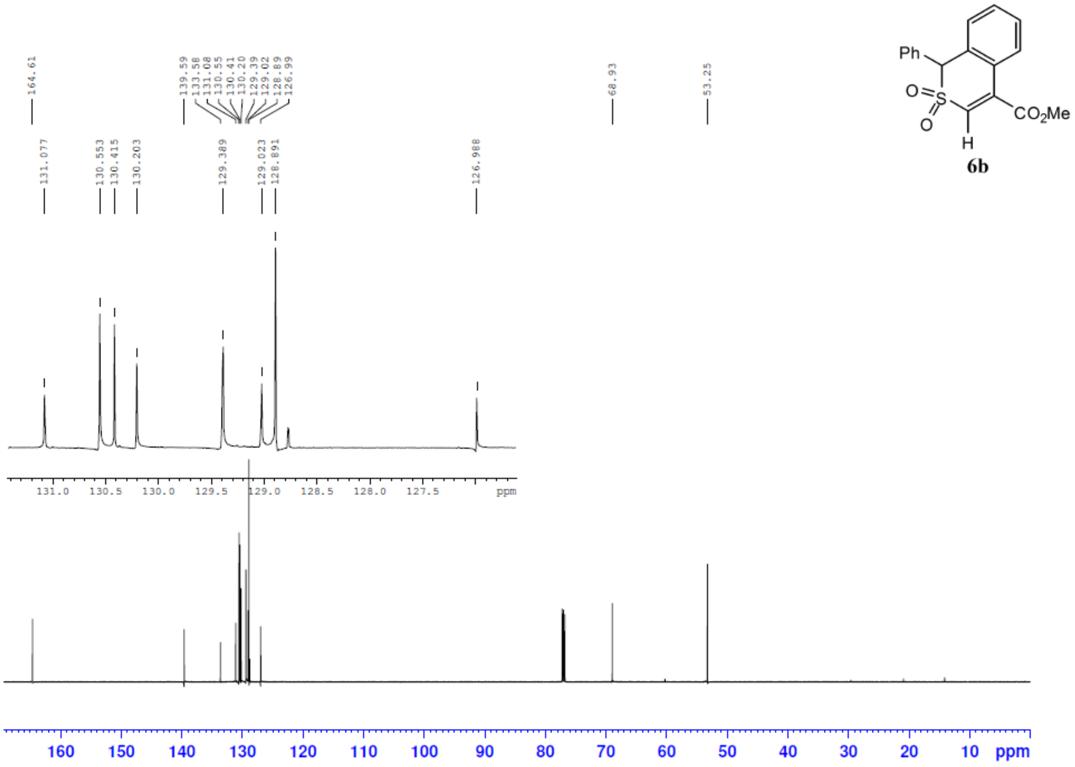

Figure S20:. The ^{13}C NMR spectrum of compound **6b**.

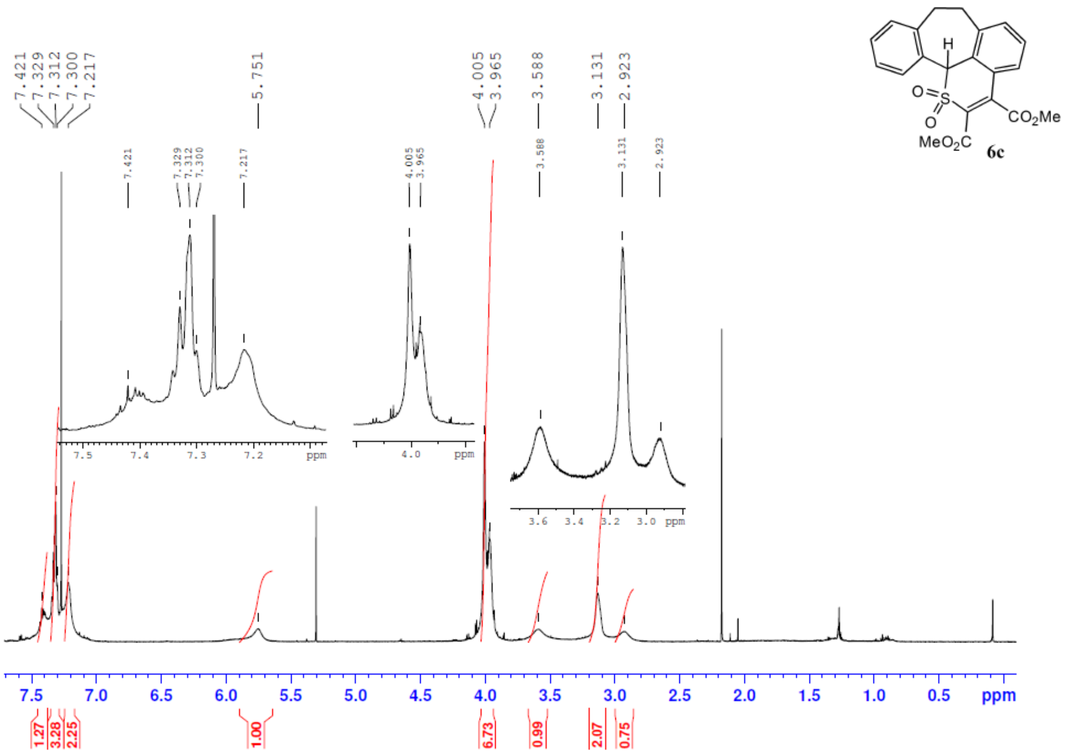

Figure S21: The ^{1}H NMR spectrum of compound **6c**.

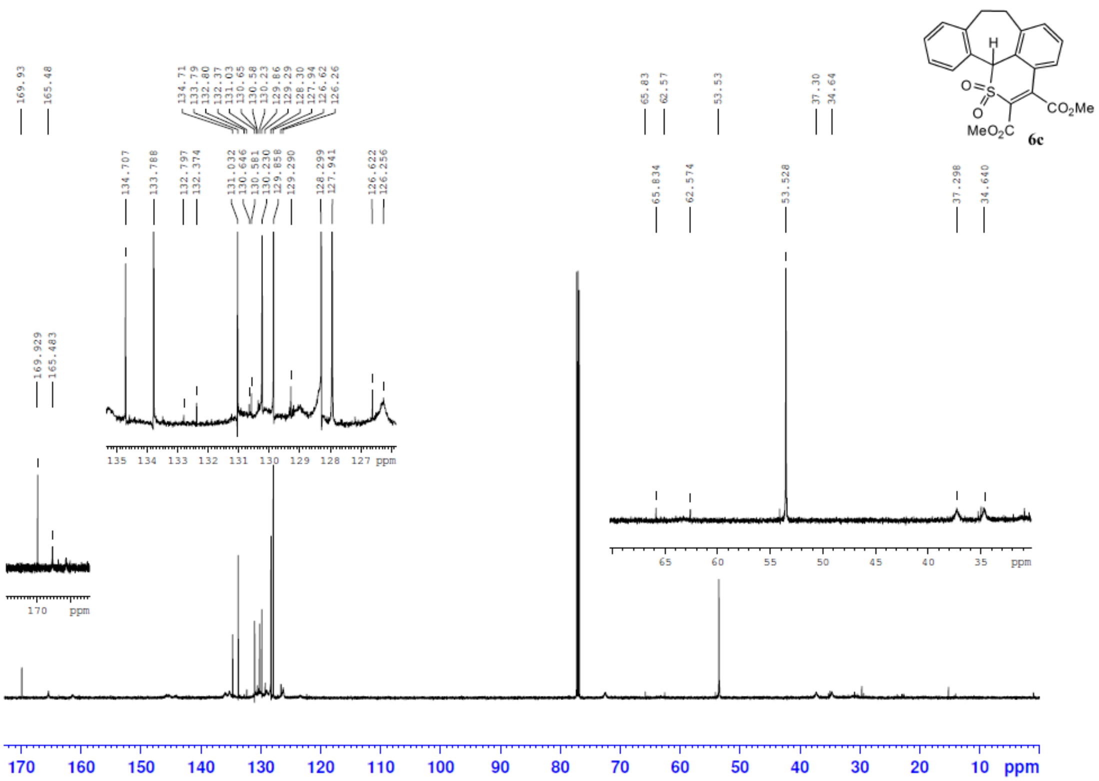

Figure S22: The ^{13}C NMR spectrum of compound **6c**.

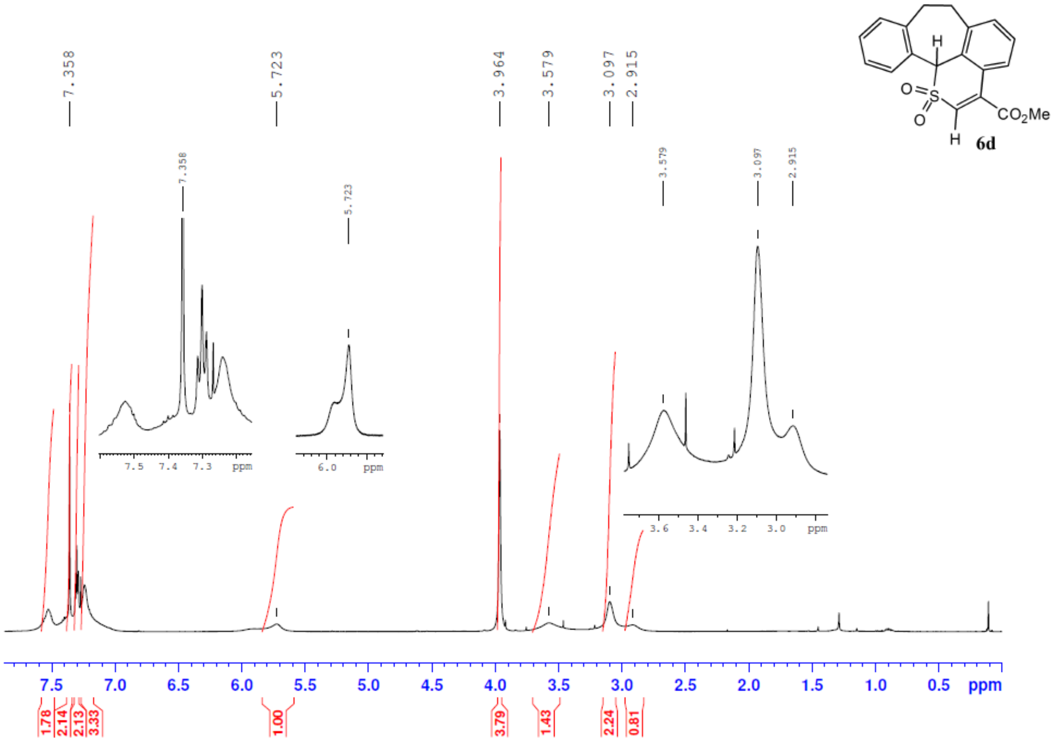

Figure S23: The ^{1}H NMR spectrum of compound **6d**.

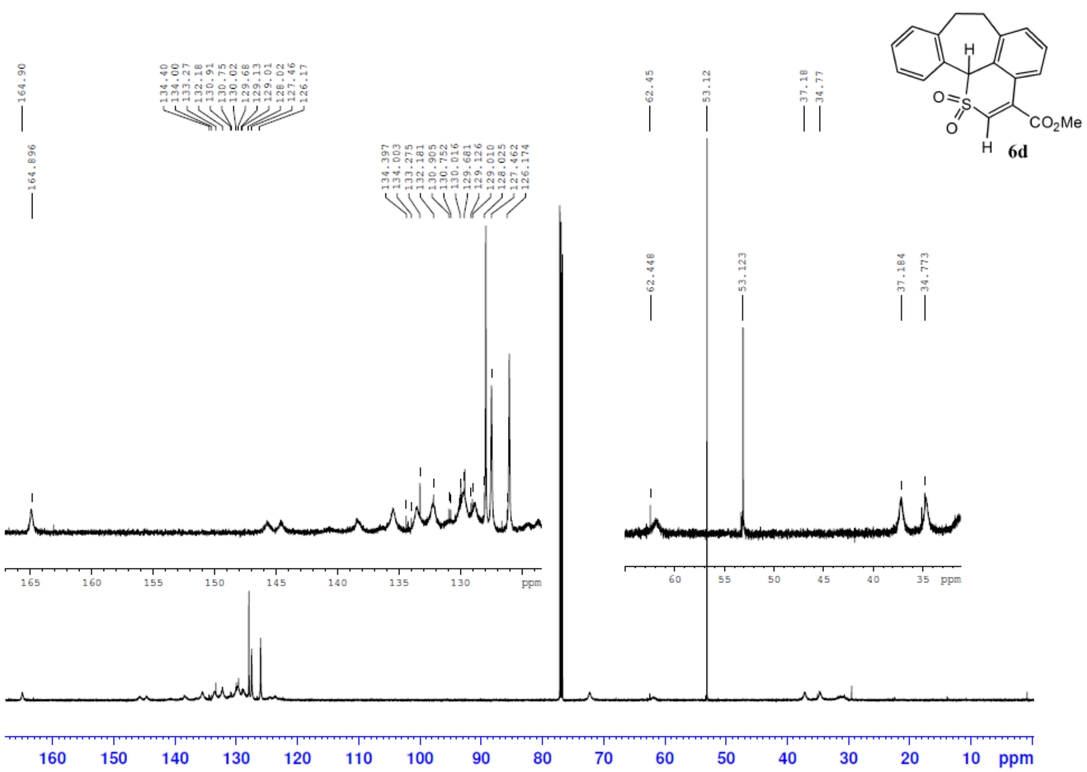

Figure S24: The ^{13}C NMR spectrum of compound **6d**.
